# A substitute variety for agronomically and medicinally important *Serenoa repens* (saw palmetto)

**DOI:** 10.1038/s41598-019-41150-z

**Published:** 2019-03-18

**Authors:** Yogini Jaiswal, Daniel Weber, Aaron Yerke, Yanling Xue, Danielle Lehman, Taufika Williams, Tiqiao Xiao, Daniel Haddad, Leonard Williams

**Affiliations:** 1 Center for Excellence in Post Harvest Technologies, North Carolina Agricultural and Technical State University, The North Carolina Research Campus, 500 Laureate Way, Kannapolis, NC 28081 USA; 2 Fraunhofer Development Centre X-ray Technology EZRT, a Division of Fraunhofer Institute for Integrated Circuits IIS, Department Magnetic Resonance and X-ray Imaging MRB, Am Hubland D-97074, Wuerzburg, Germany; 3 Department of Bioinformatics and Genomics, University of North Carolina at Charlotte, Charlotte, North Carolina 28223 USA; 4 Shanghai Advanced Research Institute, Chinese Academy of Sciences, Pudong District, Shanghai 201203 P. R. China; 5 Mass Spectrometry Facility, Department of Chemistry, North Carolina State University 2620 Yarbrough Drive, Campus Box 8204, Raleigh, NC 27695 USA; 6 Shanghai Institute of Applied Physics, Chinese Academy of Sciences, Pudong District, Shanghai 201203 P. R. China

## Abstract

*Serenoa repens* (saw palmetto) berries are one of the most consumed medicinal herbs in the United States and the wild green variety is used in the initial therapy of benign prostatic hyperplasia (BPH), globally. Use of saw palmetto is approved by the German Commission E, and several clinical trials are underway for evaluation of its efficacy. Exploitation of its habitats and over foraging imperil this plant, which only grows in the wild. This is the first study, to propose the use of the *S*. *repens* forma *glauca* (silver variety) as a qualitative substitute for the wild variety, to support its conservation. We compared tissue microstructures and lipid and water distribution through spatial imaging and examined metabolite distribution of three tissue domains and whole berries. This combined approach of 3D imaging and metabolomics provides a new strategy for studying phenotypic traits and metabolite synthesis of closely related plant varieties.

## Introduction

*Serenoa repens* (W. Bartram) Small [Arecaceae], commonly known as ‘saw palmetto’ is the third top-most selling herb for dietary supplements in the USA. A bulletin from American Botanical Council states that, the sales of saw palmetto in 2015 were about $23 million^1^. The berries of *S*. *repens* are the most popular herb used in therapy of benign prostatic hyperplasia (BPH) and lower urinary tract symptoms (LUTS), worldwide^2^. Their use by American Indians, in treating prostrate inflammation has been documented since 1700s and traditionally they were used to treat testicular atrophy, erectile dysfunction and oliguria^3,4^. Clinical trials are underway to assess the clinical efficacy of *S*. *repens* as a drug for BPH and prostate cancer, which are the most prevalent morbidity causes in middle-aged and older males^5–10^. Use of *S*. *repen*s formulations for treatment of BPH in stage I and II has been approved by the German Commission E^11^. Adverse drug reactions (ADRs) have been reported for *S*. *repens*. However, sources of such ADRs and their validity are cautioned by the World Health Organization^2^.

Currently, *S*. *repens* is not an endangered species, but considering the present exploitation of its natural habitats, there is a rising need to protect and prioritise its sustainability^12^. It is labelled as the “keystone species” in the Florida ecosystem and it is the major food source for state-threatened and endangered Florida black bear and panther species^13^.

It is imperative that measures be taken to conserve the wild *S*. *repens* from being endangered and extinct, at this critical juncture where its natural habitats are uncontrollably exploited. Identifying varieties of *S*. *repens* that can serve as a qualitative and quantitative substitute, can reduce the burden of exploitation of its natural habitats and protect the dependent ecological species from extinction. Silver saw palmetto, *S*. *repens* forma *glauca* is an ornamental variety of wild green *S*. *repens* (W. Bartram)^14^. The silver saw palmetto variety is easy to cultivate in containers or open land. These plants multiply by division, and the seeds germinate within 2–4 months. They are clonal, and the trunks are procumbent in nature, forming secondary roots that help in propagation and growth of the plant. Both the varieties have a good tolerance to pests, diseases, drought, soil-salt and fire, and can grow well in partly sunny or shady areas with no maintenance^15–17^. Comparison of the leaf structures of silver saw palmetto with wild green variety is been reported^16^. The berries of *S*. *repens* are used for formulation of herbal medicines and supplements, and are reported to contain phytosterols and fatty acids as the abundant bio-active phytoconstituents^18–20^. Till date there have been no studies published, comparing the secondary metabolite composition and morphological attributes of berries of the silver saw palmetto (SL) variety with wild saw palmetto green (SP) variety.

The aims of this study were, to identify the differences in morphological characteristics, global and tissue specific metabolite profiles, and spatial distribution of metabolites, in the berries of the silver and green varieties of *S*. *repens*. With a combined application of complementary 3D imaging and metabolomics methodologies, a candidate variety is recommended as a qualitative and quantitative substitute for, therapeutically important and highly exploited wild green variety of *S*. *repens*^21,22^.

## Results

### Evaluation of 3D morphometric characteristics

**Figure 1:**
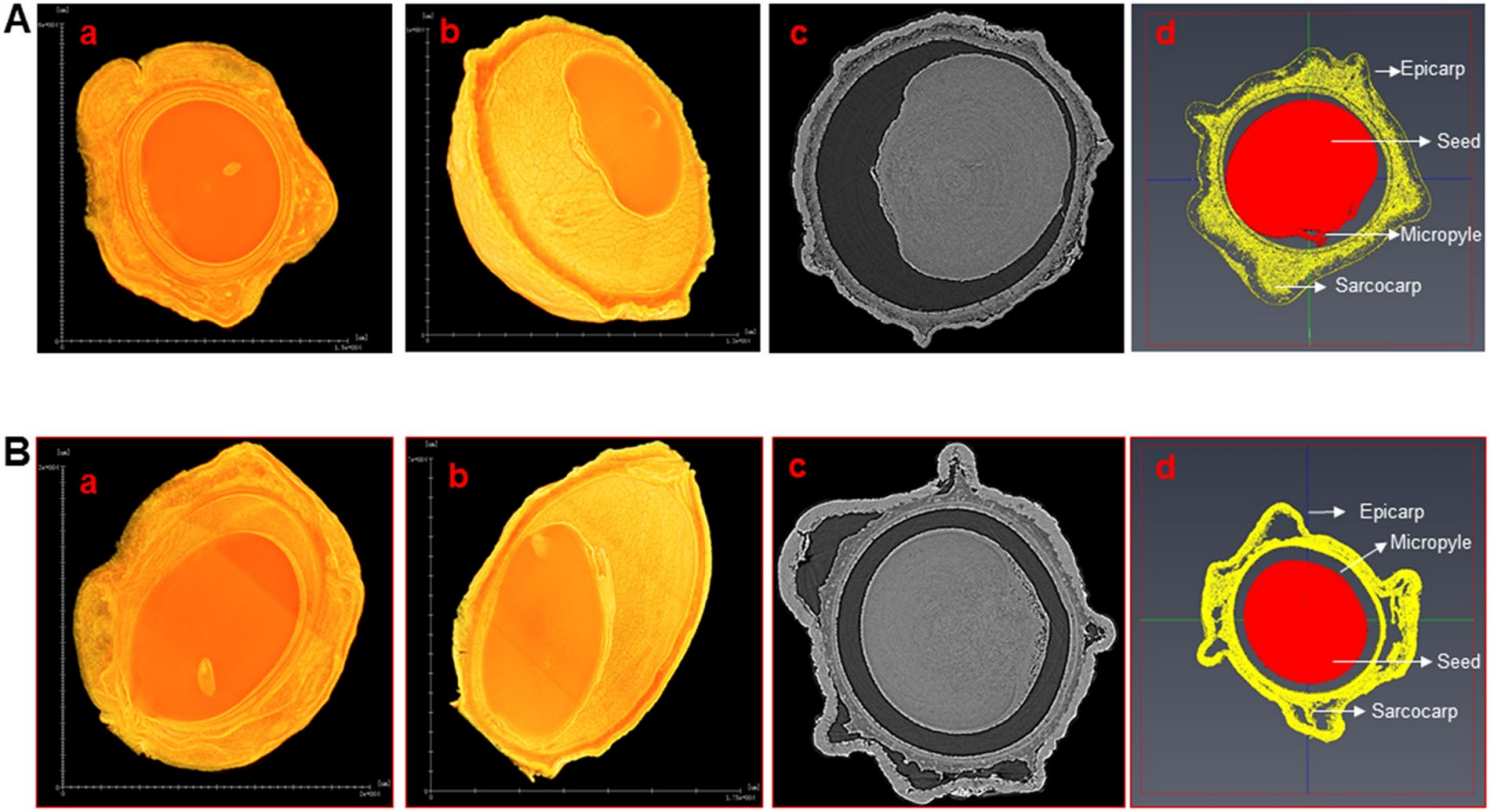
X-ray μ-CT sections and segmented regions of berries. (**A**) Berries of saw palmetto green (SP) and (**B**), berries of silver saw palmetto (SL). a, Transverse sections, b, longitudinal sections, c, micro-CT grey scale image and d, segmented regions of the berries for analysis.

The *in-vivo* structural characteristics consisting of the internal dark regions (air core), seed, epicarp, endocarp and sarcocarp were observed in the high-resolution images of saw palmetto berries acquired through X-ray computed microtomography using synchrotron radiation (SR-μCT). The transverse sections and the manually segmented regions used for automated image processing of representative berries from each variety, are shown in Fig. 1A,B. Bright pixels were observed in different regions of the berries where the vascular bundles and dense tissues attenuate the X-rays. The attenuation of X-rays is caused due to the water content in the vascular bundles and the thickness of other tissues. High resolution images of whole structure of both the varieties of the berries were obtained by stacking the virtual slices (Supplementary Files Movies S1 and S2). These 3D construction images provide an overview of the global vascular structures of the berries.

The epicarp constituting the external surface of the ellipsoidal berries appeared wrinkled and enclosed the vascular network rich sarcocarp. The endocarp appeared unilocular with a single anatropous seed marked on the raphe side by a micropyle forming a minor projection. The dark grey regions interspersed between the sarcocarp tissue and in between the endocarp and the seed, were the gas filled regions that provide porosity to the berries.

Porosity has a direct correlation with effective oxygen diffusivity, voids shape and surface area. It has an indirect correlation with the branching number and the total number of voids^23,24^. In literature, the Effective Medium Theory (EMT) and Maxwell-type structure models are used to compute effective oxygen diffusivity based on porosity^25^. Thus, porosity can be used as an indicator of metabolic processes that depend on oxygen diffusivity and affect post-harvest quality factors such as browning, formation of abnormal internal cavities in tissues and cell death^23,26^. Based on the morphometric parameters analyzed by SR-μCT, the porosity of the fleshy tissues (epicarp and sarcocarp) of the berries of SL, were found to be higher than SP (Table 1). This indicates higher diffusivity of metabolic gases in SL berries compared to SP. The low porosity in the seed, led to insufficient image contrast for the application of porosity estimation protocol by AVIZO Fire. The protocols used for calculation of morphometric parameters for SR-microCT images and porosity visualization of samples are provided in Supplementary Files S3–S8.

**Table 1:** Structural characteristics of the berries of *S*. *repens* obtained by X-ray μ-CT analysis.

Parameters	SP	SL
Porosity of epicarp and sarcocarp (%)	19.133 ± 21.956	50.021 ± 43.323
Void volume of seed (10^8^ μm^3^)	66.600 ± 0.104	116.340 ± 0.008
Void volume of epicarp and sarcocarp (10^8^ μm^3^)	11.855 ± 0.063	4.552 ± 0.005
Void shape factor of seed	4.008 ± 5.381	1.741 ± 0.322
Void shape factor of epicarp and sarcocarp	0.632 ± 0.347	0.545 ± 0.401
Crofton perimeter of seed (10^2^ μm)	30.689 ± 25.210	590.369 ± 158.62
Crofton perimeter of epicarp and sarcocarp (10^2^ μm)	58.965 ± 30.302	96.798 ± 120.049
Anisotropy factor of seed	0.724 ± 0.038	0.730 ± 0.018
Anisotropy factor of epicarp and sarcocarp	0.448 ± 0.388	0.403 ± 0.296
Sphericity of seed	1.241 ± 0.216	1.112 ± 0.012
Sphericity of epicarp and sarcocarp	1.199 ± 0.054	1.430 ± 0.260

Sample sizes used for analysis were *n* = 3. Values mentioned above indicate mean ± standard deviations of the respective parameters. SL and SP denote the silver and wild green varieties of *S*. *repens*, respectively.

The void volume in the seeds of SL were higher than the seeds of SP, whereas the epicarp and sarcocarp of berries of SL cultivar had lower void volumes compared to SP. This indicates that SL variety berries are densely connected in the fleshy region, compared to SP berries. In literature, the void shape factor is suggested to be interpreted as turtosity^24^. The void shape factor for seeds of SL variety was lower than SP, whereas in the fleshy inner regions the void shape factor of berries from both the varieties was similar. Crofton perimeter computed through AVIZO software is used to estimate the total path length for transportation of metabolic gases^24^. The higher Crofton perimeter values in SL for both segmented regions indicate that the transport of metabolic gases through the void network is more efficient in SL berries. Sphericity is used to express the shape of voids and anisotropy factor is used to calculate the diffusion that is driven by the local curvature along the gradient direction. Anisotropy factor of the seed was higher than the epicarp and sarcocarp tissue and indicated similar values for both SL and SP varieties. The higher anisotropy value of the seed indicates that the seeds have a more distinct orientation of voids and the pathways are formed in a direction that is radial to the seed core.

SL variety has higher values of porosity, void volume in seed and total transport pathway (Crofton perimeter) that are correlated to better respiratory metabolism and oxygen diffusivity within the tissues. Thus, it is suggested that SL berries can serve as a qualitative substitute for the wild grown berries of SP.

The void spaces, tissue networks and their relationship to functions in plants, is still not extensively explored. The void space characteristics visualized in this study through non-destructive computed tomography, provides quantitative information that can be utilised further for understanding plant anatomy, physiological processes, and diseases. Several studies suggest the application of 3D imaging techniques to obtain spatio-temporal information, that can be used to link genotype to phenotype traits in plants^24,26^. This is the first study to investigate and compare the structural details of *S*. *repens* berries, for the silver and wild green varieties. Differences in tissue structures and spatial morphology between these varieties, were identified and reported in this article.

### Imaging of lipid and water distribution by Magnetic Resonance Imaging (MRI)

**Figure 2:**
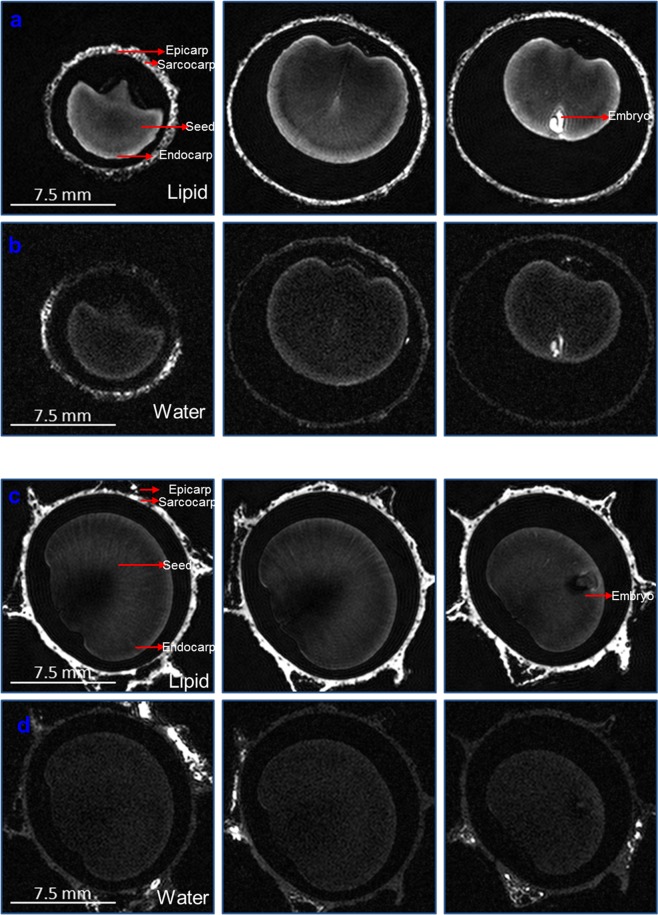
MRI images of axial slices of *S*. *repens* berries, indicating internal structures. (**a**,**b**) Represent the lipid and water distribution in wild green variety (SP), respectively and (**c**,**d**), represent the lipid and water distribution in silver variety (SL), respectively. For better visualisation of the lower intensities, a non-linear grey-scale was used which overemphasizes the lower values and chops off the highest values by setting them to the maximum value of the grey-scale.

As expected due to the relatively dry state of the whole berries and specifically the seeds, the water signal was rather weak. The lipid signal was stronger but still weak on an absolute scale. Nevertheless, in both MR images showing the water distribution and the lipid distribution, the seed could clearly be distinguished from the surrounding shell due to the enclosed air (see Fig. 2).

Furthermore, the remaining flesh on the outer shell (comprising of the epicarp and sarcocarp) also appeared bright in the MR images, especially in the lipid images, because it contained a relatively high amount of lipids and water, compared to the seed.

Substructures within the seed were clearly visible mainly in the lipid MR images. Since the water signals were much weaker than the lipid signals, it only allowed visualization of the macrostructure. In one sample a very strong signal originated from all parts of the embryo in the seed. In the other sample, the strong signal originated only from outer parts of the embryo. Since in both samples, lipid and water images showed high signal intensities, the water and lipid content in the parts that appeared bright were obviously higher than in the remaining seed. Mainly obvious in the lipid images was a bright rim of the seeds, indicating a higher lipid content in the endocarp compared to the rest of the seed. The ray-like internal substructure of the seeds can be seen in the lipid images with adjusted grey scale (see Fig. 2). Longitudinal section images of SP indicating the lipid and water distribution are shown in Supplementary File S9.

The SL berry possessed a thicker and apparently less dry fleshy sarcocarp layer compared to the SP berries. This is also visualized in the MR images which exhibit a thicker and more serrated fleshy sarcocarp layer for SL which had a stronger MR signal in both, the lipid and the water images. Again, the absolute water signal of the relatively dry berry was rather weak. The lipid signal was stronger as expected, but of course still weak on an absolute scale. Due to the enclosed air, in both, water and lipid images, the seed was clearly distinguished from the surrounding shell consisting of the epicarp and the sarcocarp.

As observed in the SP berry, the embryo in the seed of SL berry exhibited regions with increased lipid and water signals. The ray-like substructure of the seed is visible in the lipid images with adjusted grey-scale (see Fig. 2).

**Figure 3:**
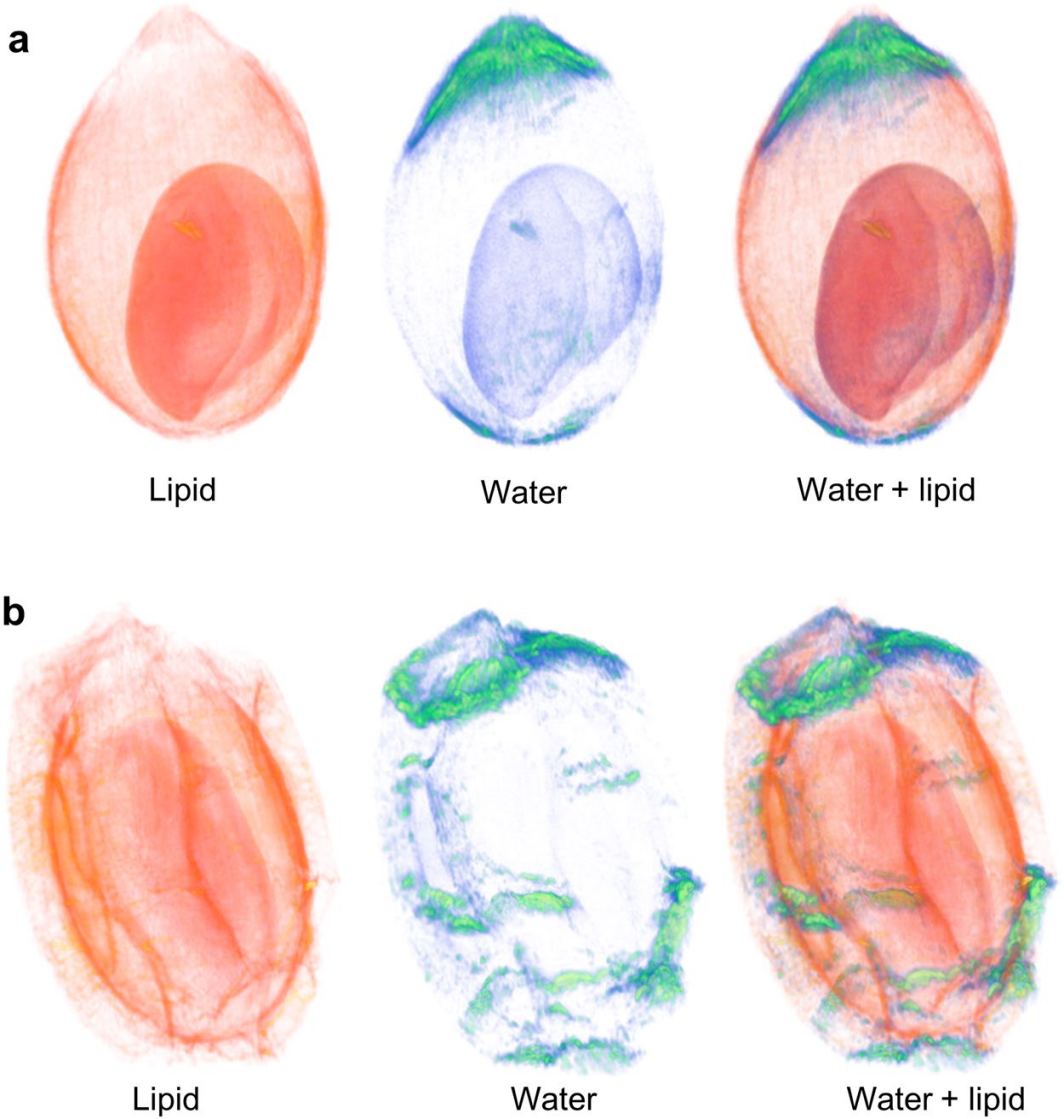
Volume rendering from 3D datasets obtained by MRI imaging of *S*. *repens* berries. (**a**) 3D volume rendering images of wild green variety (SP), and (**b**), 3D volume rendering images of silver variety (SL). The lipid, water and overlay of lipid and water distribution signals are indicated in the left, middle and right 3D images.

Volume renderings of the whole 3D dataset provide an overview of the whole berries and their lipid and water distribution (see Fig. 3). Volume renderings of seed of SL berry with the outer surface of the seed shown as solid structure for better discrimination of the seed are shown in (Supplementary File S10).

The application of non-invasive MRI technique for 3D imaging of lipid and water in this study, enabled visualization of the tissue specific distribution of the commercially and therapeutically valuable lipids of *S*. *repens*, within the whole berry. The comparative spatial analysis of the berries of the silver and wild green variety allows us to assume that the silver variety can be used as a qualitative substitute for the wild green variety of *S*. *repens*. The identification of differences in lipid distribution in various tissues provide opportunities for further investigation into the transcription factors that are responsible for lipid accumulation^27^. The reported non-invasive MRI method can be applied to study phenotypic traits, and characterization of transgenic and mutant varieties.

### Matrix assisted profiling of extracts of berries of *S*. *repens*

1,8-bis(dimethyl-amino)naphthalene (DMAN) and 9-Amino acridine (9AA) were used in matrix selection. 9AA was selected as the matrix of choice over DMAN because it facilitated better ionization of the low molecular weight metabolites, identification of more compounds, and low interference from matrix with only a few peaks above 100 Da^28^. In negative mode, 9AA exhibited a characteristic [M-H]^−^ peak at *m/z* 193.061 and proton adducts with sample metabolites, with no matrix interference. Both positive and negative mode were used for screening of ionization of extracts. However, as negative mode provided identification of more compounds of interest, it was selected for further analysis.

For identification of metabolites from the selected varieties of berries by Matrix-Assisted Laser Desorption Ionization Time-of-Flight Mass Spectrometry (MALDI-TOF/TOF MS) analysis, chloroform extracts (10 mg/mL) of five samples of each variety were used. The average percentage extractive yields with their standard deviations (*n* = 5) for chloroform extracts of SP and SL berries were 0.484 ± 0.113% and 0.693 ± 0.531%, respectively.

**Figure 4:**
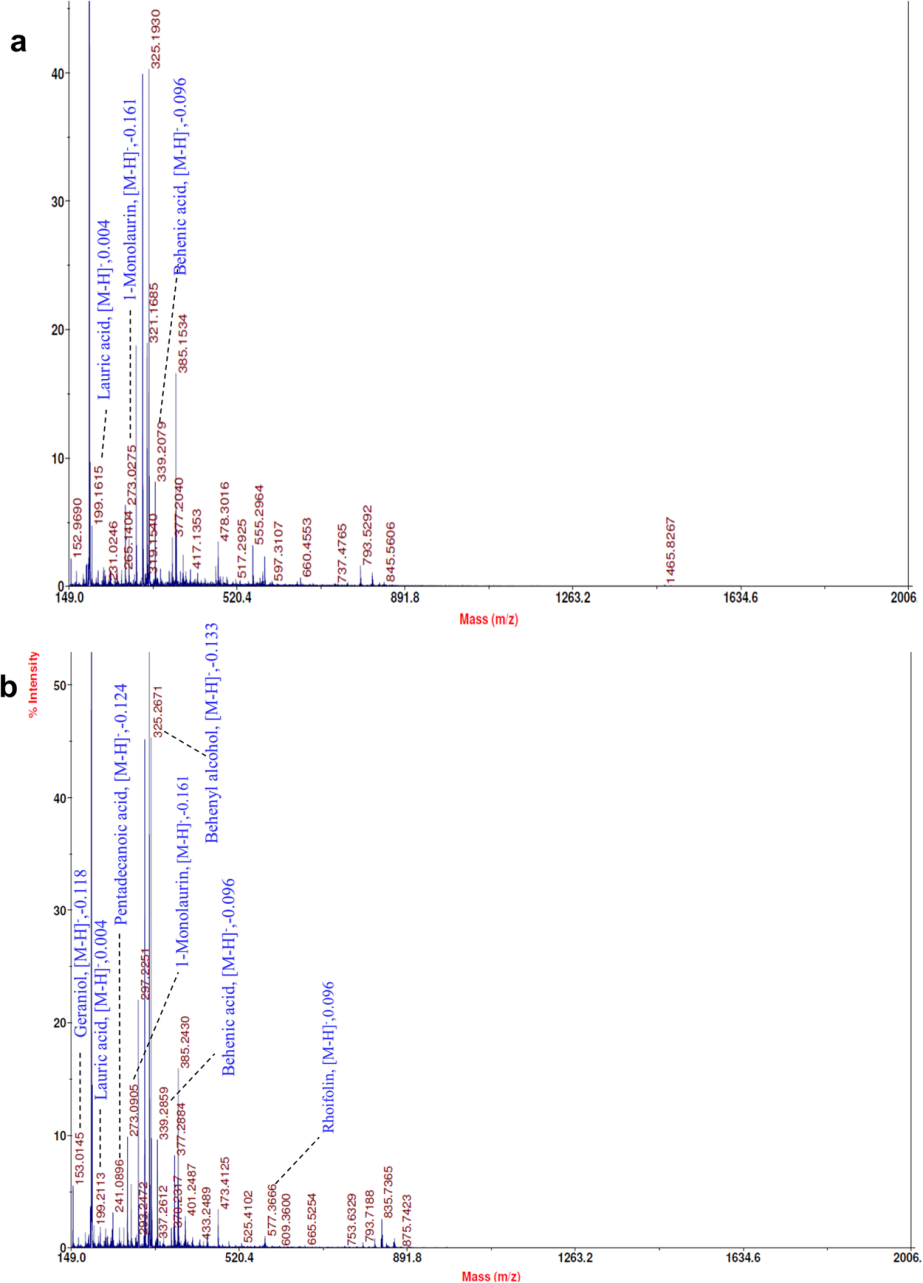
Negative mode MALDI-TOF/TOF MS spectra of selected fruit varieties. (**a**) Chloroform extract of SL variety and (**b**), chloroform extract of SP variety. Selected identified compounds are labelled with compound names and mass deviations.

The peak relative intensities were averaged for all the samples. In summary, 31 metabolites were putatively identified with [M-H]^−^ ions in the negative mode (Table 2). The identified metabolites comprised of 11 fatty acids, 3 fatty alcohols, 4 phytosterols, 2 polyprenoids, 4 flavonoids, 3 saccharides, and 4 other lipids. Of these 31 metabolites, 30 and 25 metabolites were identified in the silver SL and wild green SP variety, respectively. The commercial importance of *S*. *repens* berries and their products are due to the predominant fatty acids and phytosterols constituents^20,29^. The findings of this study, are in agreement to the reports in literature, and fatty acids and phytosterols were the most identified compounds in extracts of both selected varieties. Among the identified fatty acids, 10 were common in both varieties. The MALDI-TOF/TOF MS spectra of both the varieties were similar (Fig. 4). The qualitative variations in the global metabolite profiles of both the varieties were small, compared to the differences in the relative intensities of the common fatty acids and lipids.

**Table 2:** Metabolites putatively identified in extracts of *S*. *repens* berries by MALDI-TOF/TOF analysis in negative mode.

Constituents	Measured [M-H]^−^	Theoretical [M-H]^−^	Mass measurement accuracy (Δ ppm)	SP Relative Intensities	SL Relative Intensities	METLIN ID
**Fatty acids**						
Isomyristicin	191.080	191.071	47.10	—	1.932	90582
Hexadecanoic acid (palmitic acid)	255.227	255.233	−23.51	1.69	0.86	187
Heptadecanoic acid (margaric acid)	269.257	269.248	33.43	2.06	0.42	4206
2,3-Dihydroxypropyl dodecanoate (1-Monolaurin)	273.187	273.207	−73.20	4.89	8.06	344007
9,12-Octadecadienoic acid (linoleic acid)	279.245	279.233	42.97	1.20	0.70	191
11-Octadecenoic acid (vaccenic acid)	281.210	281.248	−135.11	1.29	1.90	3552
9-Octadecenoic acid (oleic acid)	281.244	281.248	−14.22	6.64	5.35	190
n-Octadecanoic acid (stearic acid)	283.256	283.264	−28.24	2.58	0.45	189
11-Eicosenoic acid (gondoic acid)	309.298	309.279	61.43	5.01	0.83	3554
Eicosanoic acid (arachidic acid)	311.287	311.295	−25.70	9.45	41.27	401
Docosanoic acid (behenic acid)	339.327	339.326	2.95	2.56	10.18	344007
**Fatty alcohols**						
1,20-Eicosanediol	313.322	313.311	35.11	2.86	2.84	95423
1-Docosanol (behenyl alcohol)	325.328	325.347	−58.40	9.86	37.74	4298
1-Tetracosanol (lignoceryl alcohol)	353.370	353.378	−22.64	0.8	1.31	46173
**Phytosterols**						
Campest-5-en-3beta-ol (campesterol)	399.386	399.363	57.59	0.98	0.71	167
Stigmasta-5,22-dien-3beta-ol (stigmasterol)	411.326	411.363	−89.94	—	0.33	168
Cycloarterenol (cycloartenol)	425.345	425.378	−77.58	—	0.41	34476
beta-sitosteryl-beta-D-glucopyranoside (daucosterol)	575.481	575.431	86.89	0.45	0.34	89636
**Other lipids**						
Dodecanoic acid (lauric acid)	199.174	199.170	20.08	3.74	3.06	357
5-Pentadecylresorcinol (resorcinol)	319.277	319.264	40.72	1.56	2	68597
Propyl 9-octadecenoate (propyl oleate)	323.287	323.295	−24.75	1.65	2.01	97358
23-methyl-tetracosanoic acid	377.338	377.342	−10.60	2.9	2.47	73679
**Polyprenoids**						
3,7,11,15-Tetramethylhexadec-2-en-1-ol (phytol)	295.318	295.300	60.95	1.37	0.45	391
Kaempferol 3-glucoside-7-xyloside	579.169	579.135	58.71	—	0.42	50152
**Flavonoids**						
2-Hydroxyxanthone	211.042	211.04	9.48	—	0.44	44452
2-Methoxyxanthone	225.057	225.055	8.89	—	1.4	43829
3,4′,5,7-Tetrahydroxyflavone (kaempferol)	285.037	285.040	−10.52	1.82	0.36	3410
Apigenin-7-O-rhamnoglucoside (rhoifolin)	577.152	577.156	−6.93	0.67	0.79	44401
**Saccharides**						
6-Deoxy-L-Galactose (fucose)	163.053	163.061	−49.06	2.98	—	63169
(3S,4R,5R)-1,3,4,5,6-pentahydroxyhexan-2-one (D-Fructose)	179.056	179.056	0	2.88	0.53	68675
(2R, 3R, 4R, 5R)-hexane-1,2,3,4,5,6-hexol (D-Mannitol)	181.073	181.071	11.05	5.19	0.69	142

Metabolites were putatively identified based on mass accuracy and comparison to chemical databases. SL and SP denote the silver and wild green varieties of *S*. *repens*, respectively. The relative intensities of samples were calculated with *n* = 5. (—) indicates absence of the constituent in the listed sample group.

Based on the sensitivity of the selected method, the observed discriminating features were the presence of some phytosterols that were found only in the SL variety, viz. stigmasterol (*m/z* 411.326), cycloartenol (*m/z* 425.345) and kaempferol 3-glucoside-7-xyloside (*m/z* 579.169). Also, distinctively only SP exhibited the presence of fucose (*m/z* 163.053). SL had higher intensities for arachidic acid (*m/z* 311.287), behenic acid (*m/z* 339.327), behenyl alcohol (*m/z* 325.328) and 1-monolaurin (*m/z* 273.187) compared to SP. SP showed relatively higher intensities of stearic acid (*m/z* 283.256), gondoic acid (*m/z* 309.298), margaric acid (*m/z* 269.257), fructose (*m/z* 179.056) and mannitol (*m/z* 181.073) compared to SL. The MALDI-TOF/TOF MS spectra of AA matrix is shown in Supplementary Fig. S11.

MALDI-TOF/TOF MS is a soft ionization technique that can be used for simultaneous high-throughput analysis of lower molecular weight metabolites in complex samples, with selection of an appropriate matrix. 9AA used in this study, effectively ionized low molecular weight acidic metabolites in the selected samples with no matrix interference in their mass spectral profiles. All identifications of the constituents in this method are putative based on mass accuracy of 50 ppm, and thus there exist possibilities for identifying other metabolites that may fall within the given *m/z* range. Thus, in this study MALDI- TOF/TOF MSI was not the only method used to draw conclusions for the scientific question studied. The study involves combination of sensitive and accurate hyphenated chromatography methods and imaging techniques with MALDI- TOF/TOF MSI, to complement and support the findings from this technique.

This suggests that, complemented with high sensitivity and accuracy methodologies such as Liquid chromatography–mass spectrometry (LC-MS) and Gas chromatography–mass spectrometry (GC-MS), MALDI- TOF/TOF MSI with 9AA as a matrix can be effectively used in plant research for metabolome analysis.

### MALDI-MSI visualization of secondary metabolites

**Figure 5:**
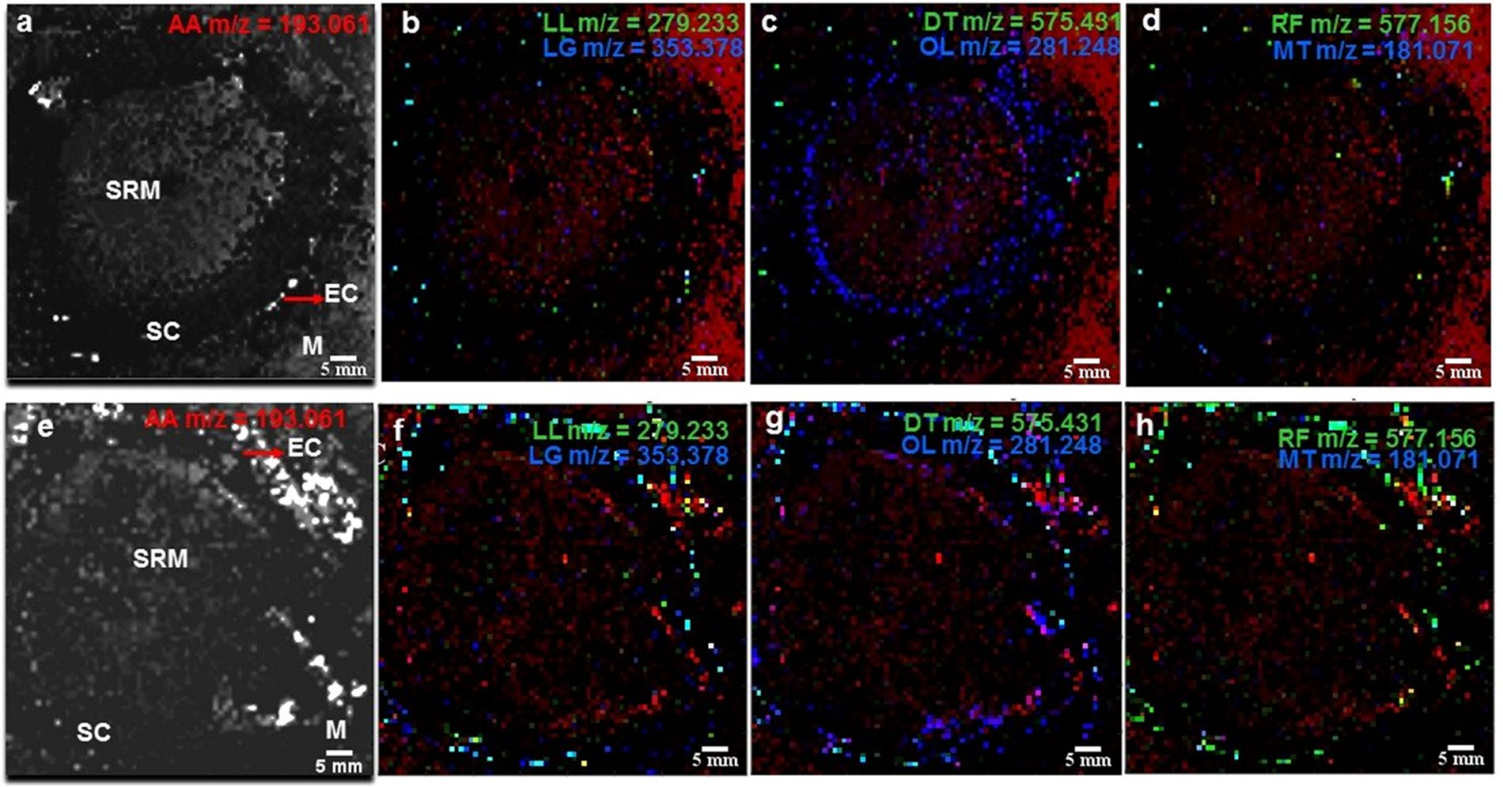
MALDI-MSI of representative metabolite distribution in transverse sections of berries of *S*. *repens* identified without TIC optimization (**a**–**d**), represent SP variety and (**e**–**h**), represent SL variety with 9-Amino acridine (AA) (red, *m/z* 193.061) as the matrix. (**a**,**e**), represent binary images of sections of SP and SL, respectively. (**b**,**f**) Depict overlay of ion images for linoleic acid (green, *m/z* 279.233) and lignoceryl alcohol (blue, *m/z* 353.378). (**c**,**g**) Depict overlay of ion images for daucosterol (green, *m/z* 575.434) and oleic acid (blue, *m/z* 281.248). (**d**,**h**) Depict overlay of ion images for rhoifolin (green, *m/z* 577.156) and mannitol (blue, *m/z* 181.071). All images were generated without TIC normalization method with a *m/z* window of 50 ppm. EC represents the epicarp, SC the sarcocarp, SRM is the region of seed location deposited by matrix and M represents the deposited matrix.

Sublimation with 9AA as the matrix was opted for imaging of transverse sections of the berries, based on its ability to ionize small molecules in negative mode and the efficiency of sublimation process^28,30^. The deposition of matrix was difficult on the seed region of the berries, like some tissues reported till date^31^. Sections of varying thickness for the seed, ranging from 10–30 µm were prepared but unlike the sarcocarp and epicarp regions, a uniform coating of the matrix was not obtained. Thus, image analysis was performed only on the sarcocarp and epicarp of the berries. In Matrix-Assisted Laser Desorption Ionization-Mass Spectrometry Imaging (MALDI-MSI), post analysis selection of a single normalization method that provides low matrix signals and optimum signals for selected ions in different varieties of samples is challenging. For image analysis, normalizations with and without total ion count (TIC) were performed (Supplementary Fig. S12)^32^. Image analysis without TIC optimization was carried out for identifying the distribution of selected phytoconstituents (see Fig. 5).

The resultant peak list generated from putative identification of metabolites by MALDI-TOF/TOF analysis was used to visualize metabolites in the tissues of berries, identify their distributions and reveal the tissue metabolic heterogeneity between SL and SP **(**Fig. 5**)**. The hollow region due to absence of seed was covered with matrix deposition. In SP and SL, the localization of linoleic acid (*m/z* 279.245) and lignoceryl alcohol (*m/z* 353.370) was found in the sarcocarp and epicarp region (Fig. 5b,f). Daucosterol (*m/z* 575.481) and 9-octadecenoic acid (*m/z* 281.244) were found to be distributed in the sarcocarp and epicarp for both SL and SP. The abundance for 9-octadecenoic acid (oleic acid) was much higher in both SL and SP, compared to the other metabolites visualized (Fig. 5c,g). Unlike the above mentioned fatty acids and alcohols, rhoifolin (*m/z* 577.152) and mannitol (*m/z* 181.072) were localized in the epicarp (Fig. 5d,h). Images for localization of each metabolite in the final peak list were generated through MSi Reader software. The intensities of selected ions in sections of berries of SL and SP varieties are depicted in Supplementary Fig. S13.

MSI combined with MALDI-TOF/TOF MS analysis in this study, provides a platform for spatial analysis of lipids and low molecular weight metabolites and studying their distribution in heterogenous plant tissues. Protocols for direct on tissue measurements, sample preparation and post-acquisition analysis, that are critical for this technique were optimised. The occurrence of metabolites in specific tissues is the result of metabolic events, tissue functions and composition. Thus, unravelling the tissue distribution of metabolites *in-situ* in plants with MALDI-MSI, suggests that it can be used as a useful imaging technology that can be coupled with other functional genomics, phenomics and metabolomics techniques.

### Macroscopic and microscopic tissue characteristics

The berries of both varieties had varied shapes ranging from ovoid, ellipsoidal, and globular, of which the ellipsoidal shape was the most common. The dimensions of five berries of each variety were measured. The average length x width were 2.0 × 1.28 cm and 1.96 × 1.32 cm for SP and SL, respectively. The berries of both varieties were dark brownish to black in colour, with a coriaceous endocarp and sarcocarp enclosing a thin hard endocarp (Supplementary Fig. S14). The seeds were anatropous, dark brown in colour, with a arillus-like appendage marking on the raphe.

Microscopy analyses of the cross sections of the berries and seeds were performed to identify their tissue characteristics. The cross sections of berries revealed similar tissue structure for both the varieties. The berries contained the epicarp, sarcocarp and the anatropous seed. The epicarp contained a single layer of dark brown epidermal cells followed by 2–3 layers of oil filled cells. The sarcocarp consisted of sclerotic cells with fine radiating pores and concentric lamellae of the wall. Oil cells containing reddish brown content were dispersed throughout the sarcocarp (Supplementary Fig. S15). The seeds contained epicarp made of epidermal cells as the outermost layer followed by large parenchyma cells and the perisperm cells. The endosperm consisted of thick-walled parenchyma cells (Supplementary Fig. S16).

Study of the morphological and anatomical details of the plant tissues is vital for understanding the functional and metabolomic implications of these structures in plants. Macroscopic and microscopic tissue identification complements the non-invasive 3D imaging techniques used in this study for investigating structural characteristics of berries of *S*. *repens*.

### Tissue specific and global metabolite analysis

Laser microdissection (LMD) of the epicarp, sarcocarp and the seeds of berries of *S*. *repens* was carried out. LC-MS and GC-MS analyses were used to identify the metabolite profiles of the isolated tissues. The analyses of extracts of whole berries and isolated LMD tissues by GC-MS revealed the presence of fatty acids and phenols. The GC-MS chromatograms for LMD tissues and whole berries of each variety are shown in Figs S17–S19. A list of identified metabolites with their retention times and Kovat’s retention indices is presented in Supplementary File S20. Notably, the extracts of whole berries of both the varieties had similar constituents. More constituents were identified in the epicarp and sarcocarp compared to the seed. Distinguishing characteristics were, the absence of cis-6-octadecanoic acid, methyl tetra decanoate and gamma tocopherol in the seed. Palmitoleic acid and palmitelaidic acid were found only in the seeds the SL variety. Similarly, pentacosanoic acid, squalene, gamma-tocopherol and methyl tetradecanoate were found only in the epicarp region of SL variety. As fatty acids contribute to the popularity and pharmacological effects of *S*. *repens*, four fatty acids (dodecanoic acid, myristic acid, palmitic acid and 9-octadecenoic acid) were quantified in extracts of whole berries of both the varieties. Both the varieties had comparable quantities of these fatty acids (Supplementary File S21).

LC-MS analysis of the extracts of whole berries revealed the presence of 21 secondary metabolites from both positive and negative modes. Flavonoids, fatty acids, polyprenoids, phytosterols, saccharides, vitamins and other organic compounds were identified in berries of both the varieties. Both varieties had identical metabolites in extracts of whole berries, except farnesol which was only found in the cultivar variety SL (Supplementary Figs S22–S27). Tissue specific metabolite analysis of the epicarp, sarcocarp and seed revealed that certain metabolites showed common distribution in tissues of both the varieties. Caproic acid, 1-monolaurin, riboflavin and ferulic acid were absent in the seeds of both the varieties. Markedly, 1-monolaurin was absent in the epicarp and present in the sarcocarp region of both the varieties. A list of all metabolites identified in respective tissues and whole extracts is presented in Supplementary File S28.

Tissue specific metabolite analysis reveals that the sarcocarp contains the highest number of fatty acids and flavonoids, followed by the epicarp and seed. The qualitative comparison of berries of both the varieties by GC-MS and LC-MS demonstrates that SL can be used as a qualitative substitute for SP.

Untargeted global profiling and targeted quantitation methods for LC-MS and GC-MS analysis were developed for extracts and isolated tissues of *S*. *repens*. With these approaches, comprehensive tissue specific global profiles of metabolomes of the target varieties of *S*. *repens* were established.

### Statistical analysis

**Figure 6:**
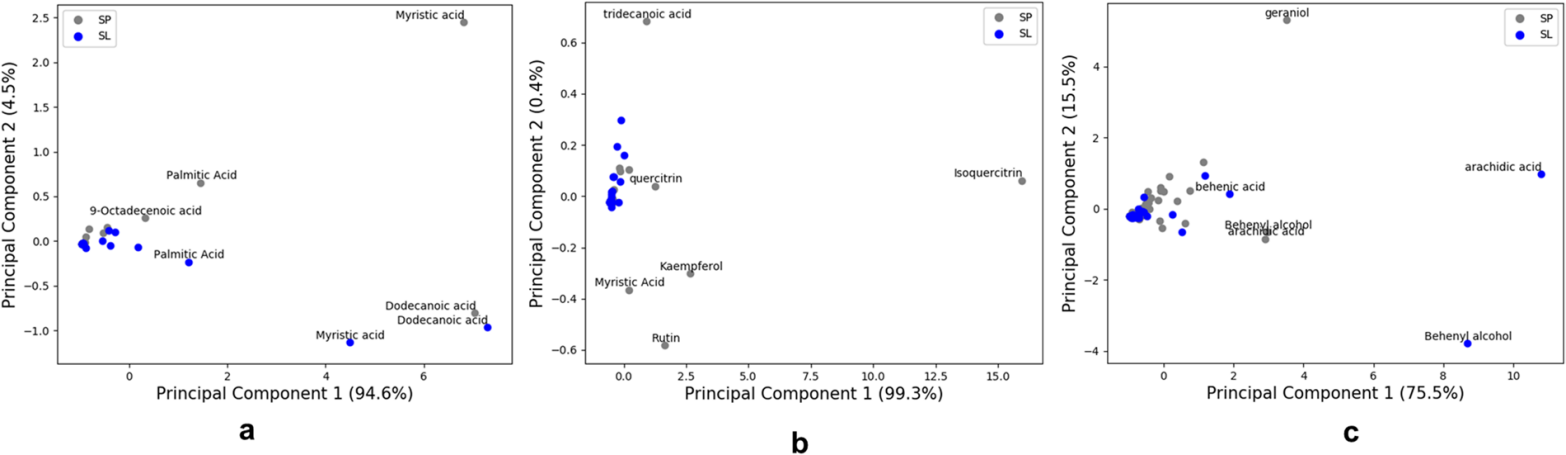
Principal component analysis plots of metabolomes of wild green and silver variety of *S*. *repens* berries. (**a**) PCA derived from all metabolites identified in GC-MS analysis (**b**) PCA derived from all metabolites identified in LC-MS analysis and (**c**) PCA derived from selected metabolites identified in MALDI-TOF analysis.

In the Principal Component Analysis (PCA) plots, the metabolites are plotted as variables and the wild and silver varieties of *S*. *repens* as groups (Fig. 6). For GC-MS, LC-MS and MALDI-TOF analysis the first principal component (PC1) contributes to the highest variance of 94.6%, 99.3% and 75.5%, respectively. The PCA plots Fig. 6a,c indicate that the two varieties are very similar to each other with respect to the phytosterols and fatty acids^33^. However, for the non-polar metabolites such as specific flavonoids, saccharides and polyprenoids the two varieties are not very similar in their metabolite profiles (Fig. 6b) and need further investigation to support SL as a substitute for SP, based on these non-polar constituents. These visualization plots, attest to the conclusions obtained from the other techniques discussed in this study. The relative abundances of the metabolites and the significantly different metabolites in both the varieties of *S*. *repens*, are illustrated in box and whiskers plots in Supplementary Figs 29–31 and Supplementary Tables 1–3. The box and whiskers plot indicate that the metabolites detected by GC-MS and MALDI-TOF analysis are not significantly different. Whereas, for metabolites detected by LC-MS methods certain glucoside flavonoids and sugars are significantly different in their abundance between the two varieties.

## Discussion

In the present study, a comprehensive integrated approach of 3D imaging and hyphenated mass spectrometry techniques was applied, to compare and validate the substitution of the silver variety to the wild green variety of *S*. *repens*. The medicinal properties of the wild green variety are reported to be due to the phytosterols and fatty acids content. Results in this study demonstrate that, with similar metabolite compositions and contents of lipids and phytosterols, the silver variety of *S*. *repens* can serve as a qualitative substitute for its wild green variety. This is the first study to report the investigation and comparison of the berries of wild green and silver varieties of *S*. *repens*. The study further merits the investigation of differences in reproductive output, physiological performance under varying environmental conditions and stress tolerance, which are characteristics reported to be superior in most cultivated varieties compared to the wild varieties^34,35^. The results of the study also open avenues for *in-vitro* and *in-vivo* studies to compare differences in medicinal benefits of both these varieties.

The plant faces a threat to be endangered, because of the uncontrolled harvesting of its natural habitats accelerated by its high consumer demands, as it is the only herb available in first-line treatment of BPH and prostate cancer. It is imperative that steps should be taken to protect this economically and therapeutically important plant. Thus, identification of a qualitative and quantitative substitute variety will help meet consumer demands, support conservation, and restrict further exploitation of the wild grown variety.

## Methods

### SR-μCT analysis

Comparison of 3D morphology with image acquisition and analysis of berries of two varieties of *S*. *repens*, was carried out with SR-μCT analysis. The SR-μCT analyses were performed with a BL13W1 X-ray imaging and biomedical application beamline at Shanghai Synchrotron Radiation Facility (SSRF), a medium energy 3^rd^ generation SR facility operating at 3.5 GeV electron beam energy and beam current of 250 mA^36^. From each variety, five samples which provided the most representation of the morphological characteristics, were analysed. Samples of berries which were decayed or showed appearance of any ruptures or damage were excluded from analysis. For the data analysis, three datasets from the five samples analysed were selected. The samples were mounted on a rotary stage for exposure to SR light during experiment. The scans were performed by SR light beam transmission through the samples with an effective energy of 18 keV, and pixel size of 6.5 µm and 9 µm according to the different sizes of samples. The CT scan parameters applied during acquisition are: exposure time (sec) = 0.0035 for SP and 0.005 for SL, beam energy 18 keV, pixel size 6.5 (μm) for SP and 9.0 μm for SL, with the distance between CCD and sample as 40 cm and 80 cm. Projection images were recorded with a 180° rotation steps and total of 1080 projections were recorded. The obtained data were reconstructed using an in-house developed software – PITRE^37^. This software was successfully applied for analysis of several plants studied at the Shanghai Synchrotron Radiation Facility^38,39^. The image processing parameters for filtering, segmentation, calculation of Volume of Interest (VOI), porosity and void volume calculation are detailed in Supplementary File 32.

### MRI imaging and data processing

MRI of both fruit varieties was performed at the Magnetic Resonance and X-ray Imaging department of the Fraunhofer Institute for Integrated Circuits in Germany. The MRI measurements were performed using an Avance 500 MR spectrometer (Bruker, Germany) with a magnetic field strength of 11.7 T and a 15 mm birdcage coil.

Two fruit samples of the wild saw palmetto (SP) and one fruit sample of the silver saw palmetto (SL) variety were scanned. Structurally intact samples were used for the measurements and the acquired 3D datasets were subsequently analysed. For the measurements, the dry samples were placed in a sample tube and scanned at room temperature. Following MRI experiment parameters were used: 3D spin echo experiment, TE = 7 ms, TR = 2000 ms, field of view = 22.5 × 15.0 × 15.0 mm³ with a nominal spatial resolution = 100 µm isotropic, averages = 2 or 10. With each experiment one image showing mainly the water distribution and one image showing mainly the lipid distribution in the sample were acquired. Frequency selective excitation and acquisition with a frequency separation of 1750 Hz were used.

For data processing, each 3D dataset was zero filled by a factor of 2 and Fourier transformed to generate grey-scale MR images. These images were subsequently analysed with the AMIRA graphics package (FEI, Thermo Fisher Scientific, USA). 3D volume renderings were performed based on signal intensities. Like this, the outer shell of the berries could be displayed separated from the enclosed air and the seed.

### MALDI-TOF/TOF MS analysis of extracts

The “soft ionisation” ability of MALDI-TOF in detecting low molecular weight compounds coupled with low interference AA matrix, was used for fingerprinting of secondary metabolites from extracts of berries of both varieties of *S*. *repens*. Five sample sets of each variety were purchased from different commercial vendors. The samples were authenticated by the US Botanical Safety Laboratory (USBSL), of the North Carolina Arboretum, North Carolina, USA and voucher nos. 1467a and 1467b were obtained for SP and SL, respectively. Extraction of the powdered berries was carried out with a mixture of chloroform and methanol as per the procedure described by Dyer, *et al*.^40^. The prepared extracts were flushed with nitrogen and stored at −20 °C until analysis. The samples were analysed on an AB Sciex 5800 MALDI-TOF/TOF MS instrument in negative mode over the *m/z* 150–2000 mass range. Details of sample application and instrument settings are provided in Supplementary File S33.

### MALDI Mass Spectrometry Imaging of tissue sections

Transverse sections of multiple berries of both the varieties were prepared using a Leica RM 2245 microtome at a thickness of 15 μm. One tissue section of each variety which provided good characteristics for imaging, was used for image analysis. Matrix deposition on the sections was carried out by 9-AA sublimation, as described by Svatos *et al*. with slight modifications^30^. To adhere sections to the indium tin oxide-coated conductive slides, double-sided tape (HTX technologies, USA) was used for MALDI MSI. An AB Sciex 5800 MALDI-TOF/TOF MS instrument was used for imaging. Samples were analysed in negative mode. The parameters used for analysis with the AB Sciex TOF-TOF imaging acquisition software (ver 1.0 Revision 619, 2012) were: 150 μm laser step size, 10 shots per sub-spectrum and 50 shots per spectrum, 5–500 laser pulses, reflector mode, mass range *m/z* 100–2000 Da (±0.05). Details of data processing parameters for MALDI-MSI are provided in Supplementary File S34.

### Laser microdissection, GC-MS and LC-MS analysis

Cryo-sectioning of berries of both the varieties was carried out with Leica CM 1850 cryostat. Laser micro-dissection of specific tissues (epicarp, sarcocarp and seed) was carried out with a Zeiss Palm Microbeam system (P.A.L.M. Microlaser Technologies AG, Germany) equipped with a PALMRobo (Version: V4.6.0.4) software. An Agilent 7890A GC system, coupled to an Agilent 5975C electron ionization (EI) mass selective detector (MSD) was used for GC-MS analysis of whole extracts and laser dissected tissues. For LC-MS analysis, a UPLC-QTOF MS system (ACQUITY UPLC-Quattro Premier XE MS, Waters Corp., Milford, MA) was used. Details of the sample preparation, microscopy, and instrument parameters for mass spectrometry analysis are provided in Supplementary File S35.

### Data analysis

PCA loading plots were prepared to identify the similarities and dissimilarities between the metabolites of wild green and silver varieties of *S*. *repens* and the metabolites profiles generated by by GC-MS, LC-MS and MALDI-TOF techniques. Values of averages of five biological replicates (of each variety) of *S*. *repens* berries, obtained for each technique were imported into the computing environment of Python 3.54. Data were scaled and transformed using scikit-learn^41^. Rv3.4.3 (The R foundation for statistical computing, 2017) was used for further statistical analysis. Missing values were replaced by zeros, the values were log transformed and significance between the values of the two varieties was established by applying the Welch’s t-test, Wilcoxon rank sum, and the F-statistic. All tests were two sided. False discovery rate was corrected using the Benjamini Hochberg method for the t-test. Raw scores of experimental groups for each variety, obtained from GC-MS, LC-MS and MALDI-TOF techniques were used to generate box-and-whisker plots, an asterisks (*) indicates significant Welch’s t-test values ≤ 0.05 after FDR correction. The box plots represent the areas for each of the metabolites identified with the interquartile range. The whiskers presented as error bars indicate the metabolites with their deviations from the median values within the selected interquartile range.

### Code availability

The Python script for generating the PCA plots and the R script written for comparing metabolite profiles of two varieties of *S*. *repens* by various analytical methods is available at https://github.com/palomnyk/comparison_of_two_species.

## Supplementary information

Supplementary Data

Supplementary Movie S1

Supplementary Movie S2

Supplementary Dataset - Table 1

Supplementary Dataset - Table 2

Supplementary Dataset - Table 3

## Data Availability

The 3D images and data supporting results of this study are available on request from the corresponding author. A private repository for images of MRI, SR-μCT analysis and manuscript figures can be found at https://bitbucket.org/thanky0u/plant.
