# Supplementary material for: A substitute variety for agronomically and medicinally important Serenoa repens (saw palmetto)
Source: Sci Rep. 2019 Mar 18;9:4709. doi: 10.1038/s41598-019-41150-z (PMC6423146; doi:10.1038/s41598-019-41150-z)
Supplement: Supplementary file 1 — Supplementary Data [file 41598_2019_41150_MOESM1_ESM.docx]

**Supplementary materials**

**A substitute variety for agronomically and medicinally important**

***Serenoa repens* (saw palmetto)**

**Author list:** Yogini Jaiswal*^1^, Daniel Weber^2^, Aaron Yerke^3^, Yanling Xue^4^, Danielle Lehman^5^, Taufika Williams^5^, Tiqiao Xiao^4^, Daniel Haddad^2^, Leonard Williams*^1^

| **Table of contents** | | |
| --- | --- | --- |
| **S1** | Video file for 3D construction of whole fruit structure of *S. repens* (wild green) variety by μ-CT analysis………………………….. | 4 |
| **S2** | Video file for 3D construction of whole fruit structure of *S. repens* (silver) variety by μ-CT analysis…………………… | 4 |
| **S3** | Image processing flowchart and settings for segmentation protocol of μ-CT data with Avizo software………………………. | 5 |
| **Fig. S3.1** | Screenshot of segmentation protocol used for different regions of the  berries of *S. repens* using Avizo software…………………………. | 5 |
| **Fig. S3.2** | Screenshot of island filter parameter used for noise removal in micro-CT images of the berries of *S. repens,* using Avizo software……….. | 6 |
| **Fig. S3.3** | Screenshot of segmented regions of the berries of *S. repens*, using Avizo software………………………………………………………… | 6 |
| **S4** | Image processing flowchart and settings for void volume and area calculation of μ-CT data, with Avizo software | 7 |
| **Fig. S4.1** | Screenshot of void volume and area calculation flowchart used for μ-CT images of the berries of *S. repens*, using Avizo software……….. | 7 |
| **Fig. S4.2** | Screenshot of arithmetic module and its settings used in void volume and area calculation flowchart, for μ-CT images of the berries of *S. repens*, using Avizo software…………………………………………. | 7 |
| **Fig. S4.3** | Screenshot of material statistics module used in void volume and area calculation flowchart, for μ-CT images of the berries of *S. repens*, using Avizo software…………………………………………………... | 8 |
| **S5** | Image processing flowchart and settings for surface area calculation of μ-CT data, with Avizo software | 8 |
| **Fig. S5.1** | Screenshot of surface area calculation flowchart used for μ-CT images of the berries of *S. repens*, using Avizo software…………………….. | 8 |
| **Fig. S5.2** | Screenshot of generate surface module used in surface area calculation flowchart, for μ-CT images of the berries of *S. repens*, using Avizo software……………………………………………………….. | 9 |
| **Fig. S5.3** | Screenshot of surface area volume module used in surface area calculation flowchart, for μ-CT images of the berries of *S. repens*, using Avizo software……………………………………………….. | 9 |
| **S6** | Image processing flowchart and settings for surface area calculation of μ-CT data, with Avizo software | 10 |
| **Fig. S6.1** | Screenshot of porosity calculation flowchart used for μ-CT images of the berries of *S. repens*, using Avizo software………………………. | 10 |
| **Fig. S6.2** | Screenshot of ASBMR porosity module, used in porosity estimation for μ-CT images of the berries of *S. repens*, using Avizo software……. | 11 |
| **S7** | S7 Image processing flowchart and settings for calculation of tortuosity related parameters for μ-CT data, with Avizo software | 12 |
| **Fig. S7.1** | Screenshot of flowchart for calculation of tortuosity related parameters for μ-CT images of the berries of *S. repens*, using Avizo software………………………………………………………… | 12 |
| **Fig. S7.2** | Screenshot of filter by measure module, used in tortuosity estimation for μ-CT images of the berries of *S. repens*, using Avizo software……. | 12 |
| **Fig.S8** | Porosity visualisation of berries of S.repens with Avizo software | 13 |
| **Fig. S9** | MRI images of longitudinal slices through a *S. repens* berry of wild variety (SP). | 14 |
| **Fig.S10** | Volume rendering of 3D datasets obtained by MRI analysis of berry of the silver variety (SL) of *S. repens*. | 14 |
| **Fig. S11** | 9-Amino acridine used as matrix for analysis of samples in negative mode by MALDI-TOF/TOF technique………………………………... | 15 |
| **Fig. S12** | MALDI-MSI of representative metabolite distribution in transverse sections of berries of *S. repens* identified with TIC optimisation……… | 16 |
| **Fig. S13** | MALDI-MSI images of selected metabolite ions in sections of *S. repens* berries……………………………………………………… | 17 |
| **Fig. S14** | Pictorial representation of whole berries of *S. repens*………………… | 18 |
| **Fig. S15** | Microscopic features of whole berries of *S. repens*…………………… | 19 |
| **Fig. S16** | Microscopic features of seeds of *S. repens*…………………………….. | 20 |
| **Fig. S17** | GC-MS chromatograms of laser dissected tissues of *S. repens* (wild green variety) berries …………………………………………………. | 21 |
| **Fig. S18** | GC-MS chromatograms of laser dissected tissues of *S. repens* (silver variety) berries………………………………………………………… | 22 |
| **S19** | GC-MS chromatograms of extracts of whole berries of *Serenoa repens* | 23 |
| **S20** | Metabolites identified by GC-MS analysis for berries of *S. repens*…… | 24 |
| **Fig. S21** | Concentrations of fatty acids in different varieties of berries of *S. repens,* identified by GC-MS analysis………………………………… | 25 |
| **Fig. S22** | LC-MS base peak chromatograms of laser dissected tissues of *S. repens* (wild green variety) berries in positive mode ……………………………. | 26 |
| **Fig. S23** | LC-MS base peak chromatograms of laser dissected tissues of *S. repens* (silver variety) berries in positive mode……………………………. | 27 |
| **Fig. S24** | LC-MS base peak chromatograms of extracts of whole berries of *S. repens* analysed in positive mode……………………………………. | 28 |
| **Fig. S25** | LC-MS base peak chromatograms of laser dissected tissues of *S. repens* (wild green variety) berries in negative mode ……………………… | 29 |
| **Fig. S26** | LC-MS base peak chromatograms of laser dissected tissues of *S. repens* (silver variety) berries in negative mode…………………………….. | 30 |
| **S27** | LC-MS base peak chromatograms of extracts of whole berries of *S. repens* analysed in negative mode……………………………………. | 31 |
| **S28** | Metabolites identified by LC-MS analysis of berries of *S. repens*. | 32 |
| **S29** | Box and whiskers plots of the relative abundances of metabolites identified by GC-MS analysis. | 34 |
| **S30** | Box and whiskers plots of the relative abundances of metabolites identified by LC-MS analysis. Significant metabolites are indicated with an asterisk (*). | 35 |
| **S31** | Box and whiskers plots of the relative abundances of metabolites identified by MALDI-TOF analysis. | 36 |
| **S32** | Image processing of SR-μCT image datasets……………………….. | 37 |
| **S33** | Sample application and instrument settings for MALDI-TOF/TOF-MS analysis………………………………………………………………. | 38 |
| **S34** | Data processing for MALDI-MSI……………………………………. | 39 |
| **S35** | Laser microdissection, GC-MS and LC-MS analysis………………… | 39 |
|  | References |  |
|  | **Supplementary Tables** |  |
| **Table 1** | Statistical significance between metabolites of two varieties of *S. repens* by GC-MS analysis. |  |
| **Table 2** | Statistical significance between metabolites of two varieties of *S. repens* by LC-MS analysis. |  |
| **Table 3** | Statistical significance between selected metabolites of two varieties of *S. repens* by MALDI-TOF analysis. |  |

**S1 Video file for 3D construction of whole fruit structure of *S. repens* (wild green) variety by micro-CT analysis (provided as an attachment)**

**S2 Video file for 3D construction of whole fruit structure of *S. repens* (silver) variety by micro-CT analysis (provided as an attachment)**

**S3 Image processing flowchart and settings for segmentation protocol of μ-CT data with Avizo software**


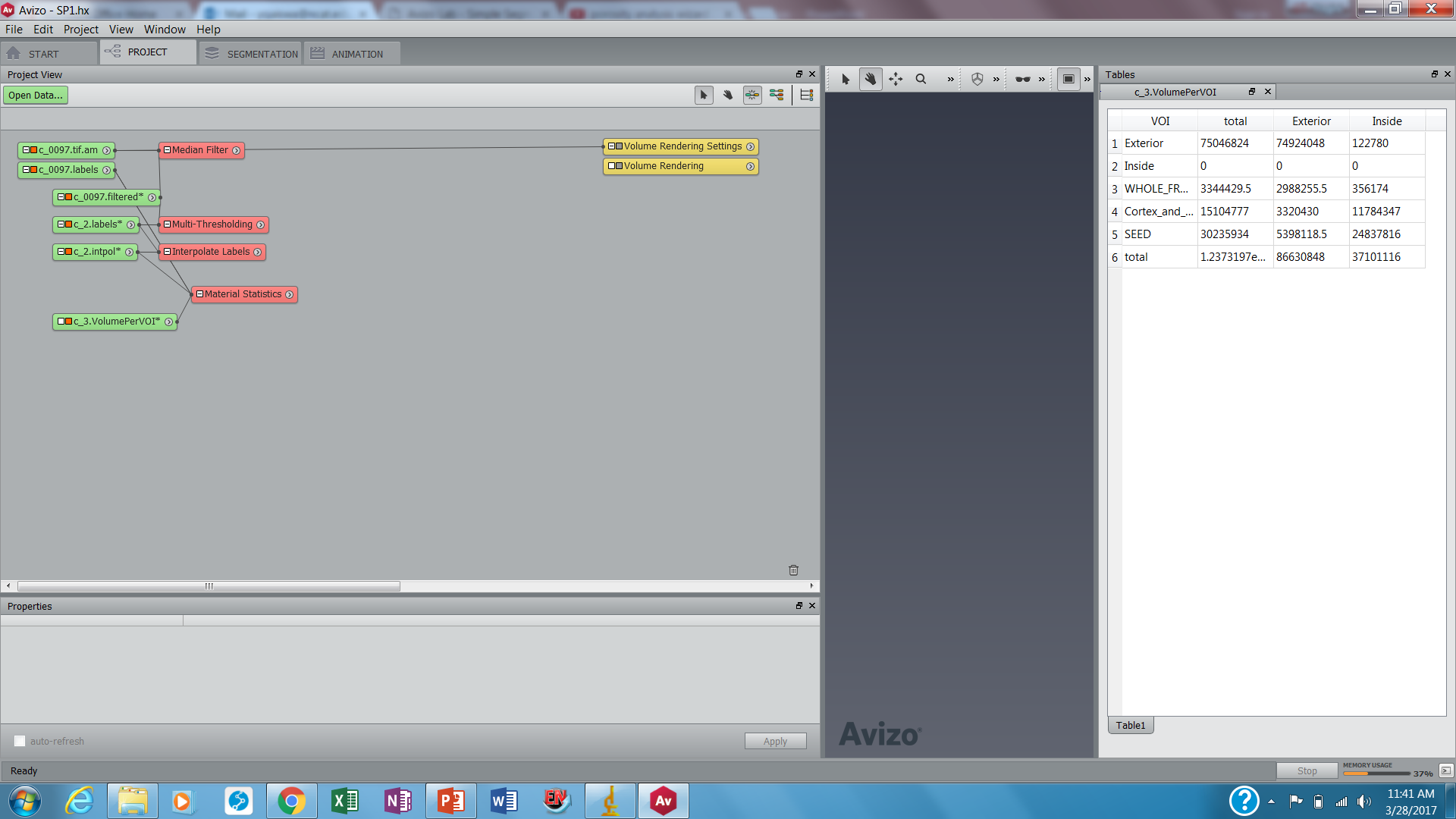


**Fig. S3.1** Screenshot of segmentation protocol used for different regions of the berries of *S. repens* using Avizo software


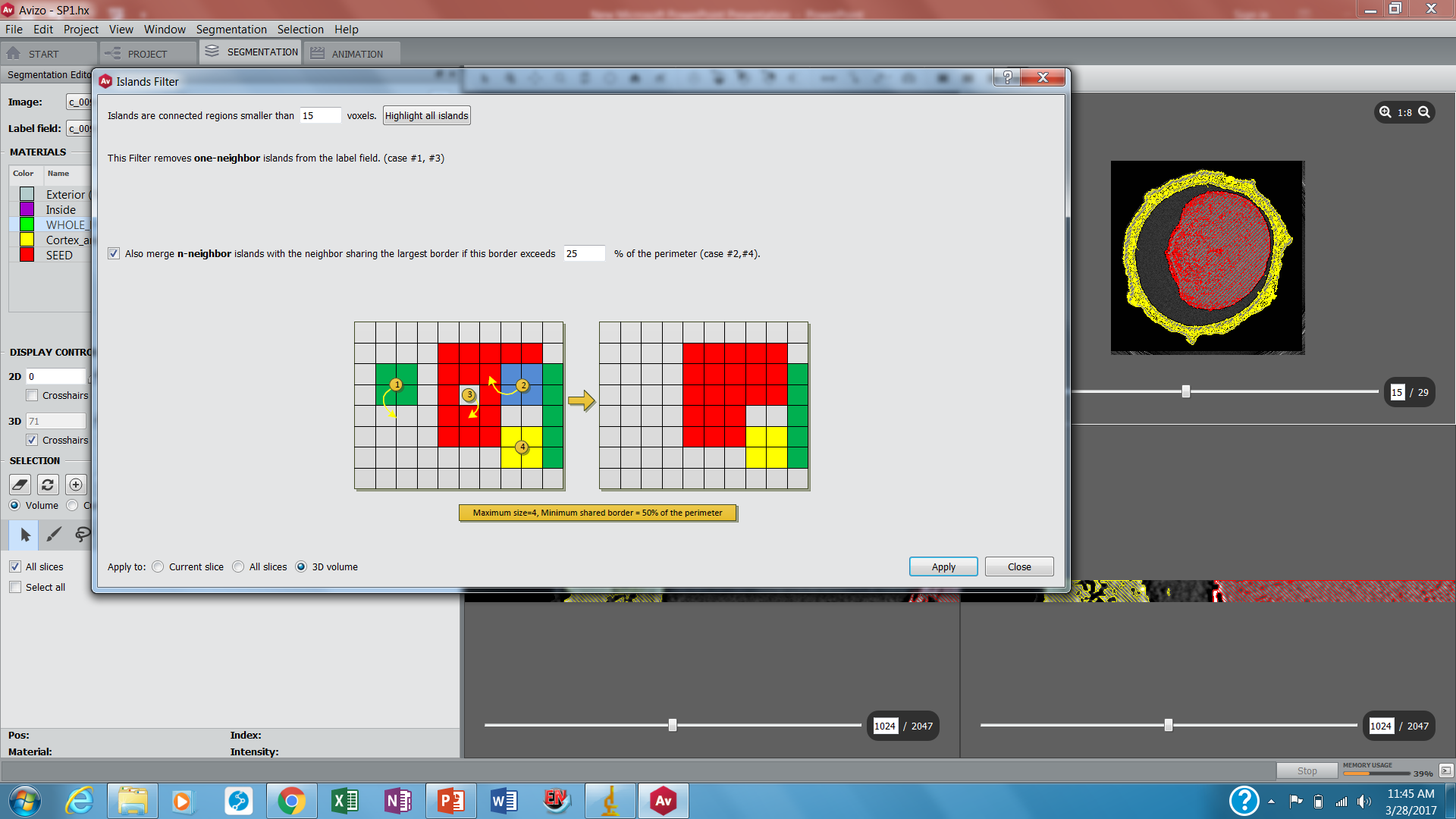


**Fig. S3.2** Screenshot of island filter parameter used for noise removal in μ-CT images of the berries of *S. repens,* using Avizo software


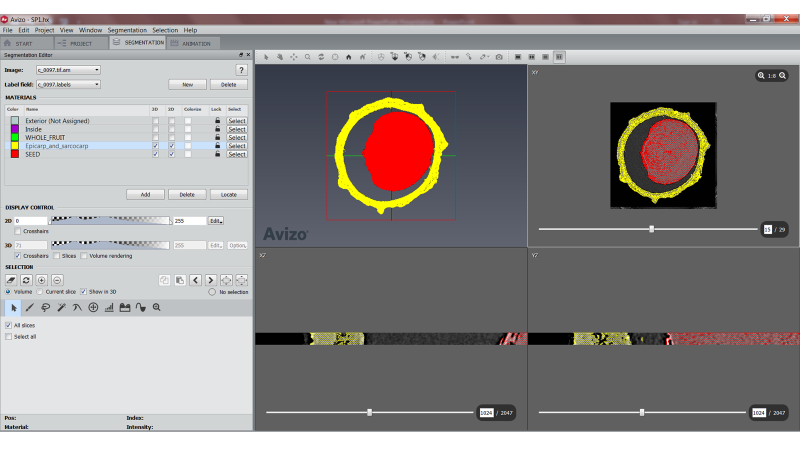


**Fig. S3.3** Screenshot of segmented regions of the berries of *S. repens*, using Avizo software. The segmented epicarp and sarcocarp region of the berries of *S. repens* are assigned yellow colour and the red segmented region indicated the seed region. The exterior and void region is represented in grey color.

**S4 Image processing flowchart and settings for void volume and area calculation of μ-CT data, with Avizo software**

**
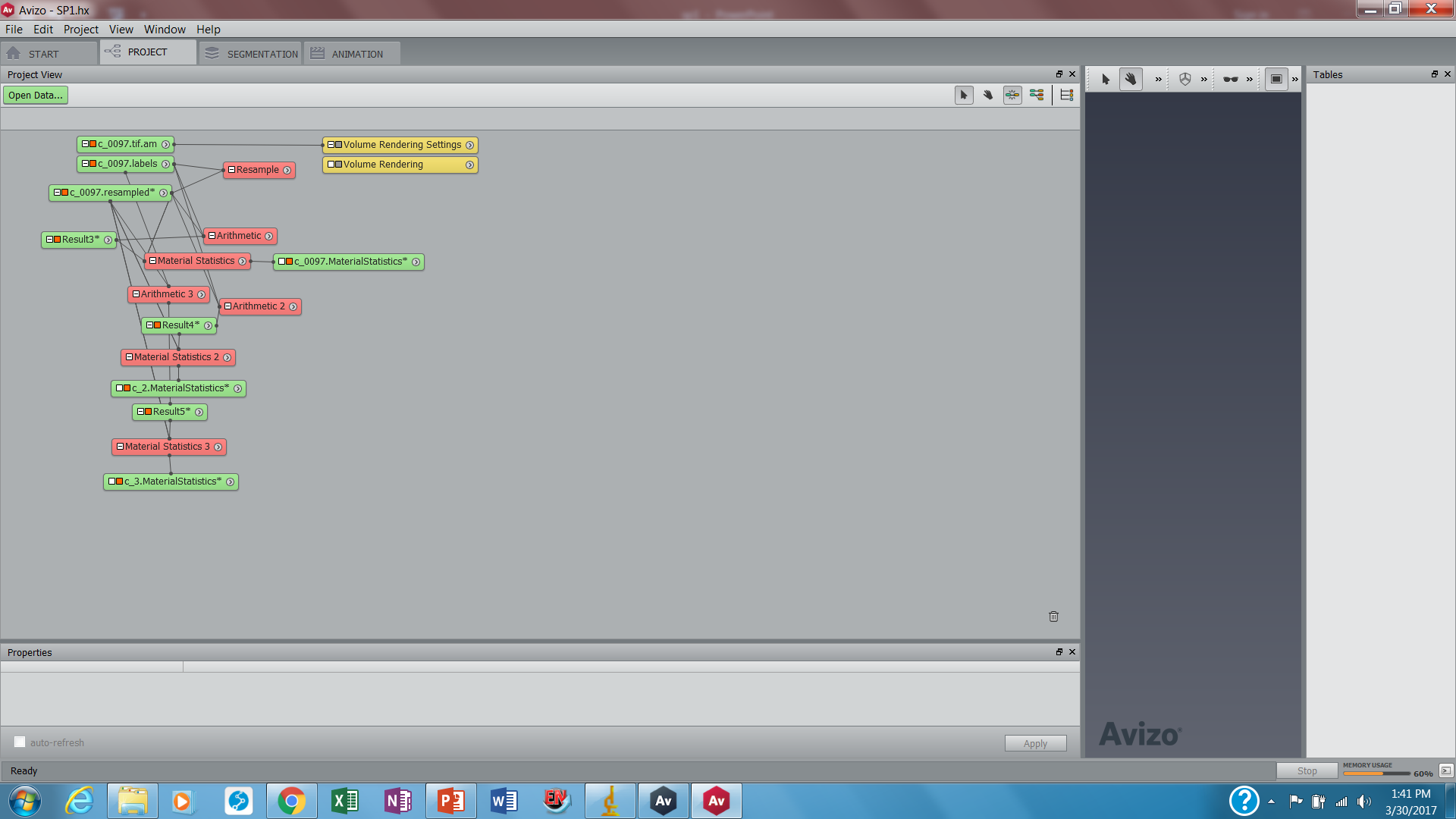
**

**Fig. S4.1** Screenshot of void volume and area calculation flowchart used for μ-CT images of the berries of *S. repens*, using Avizo software

**
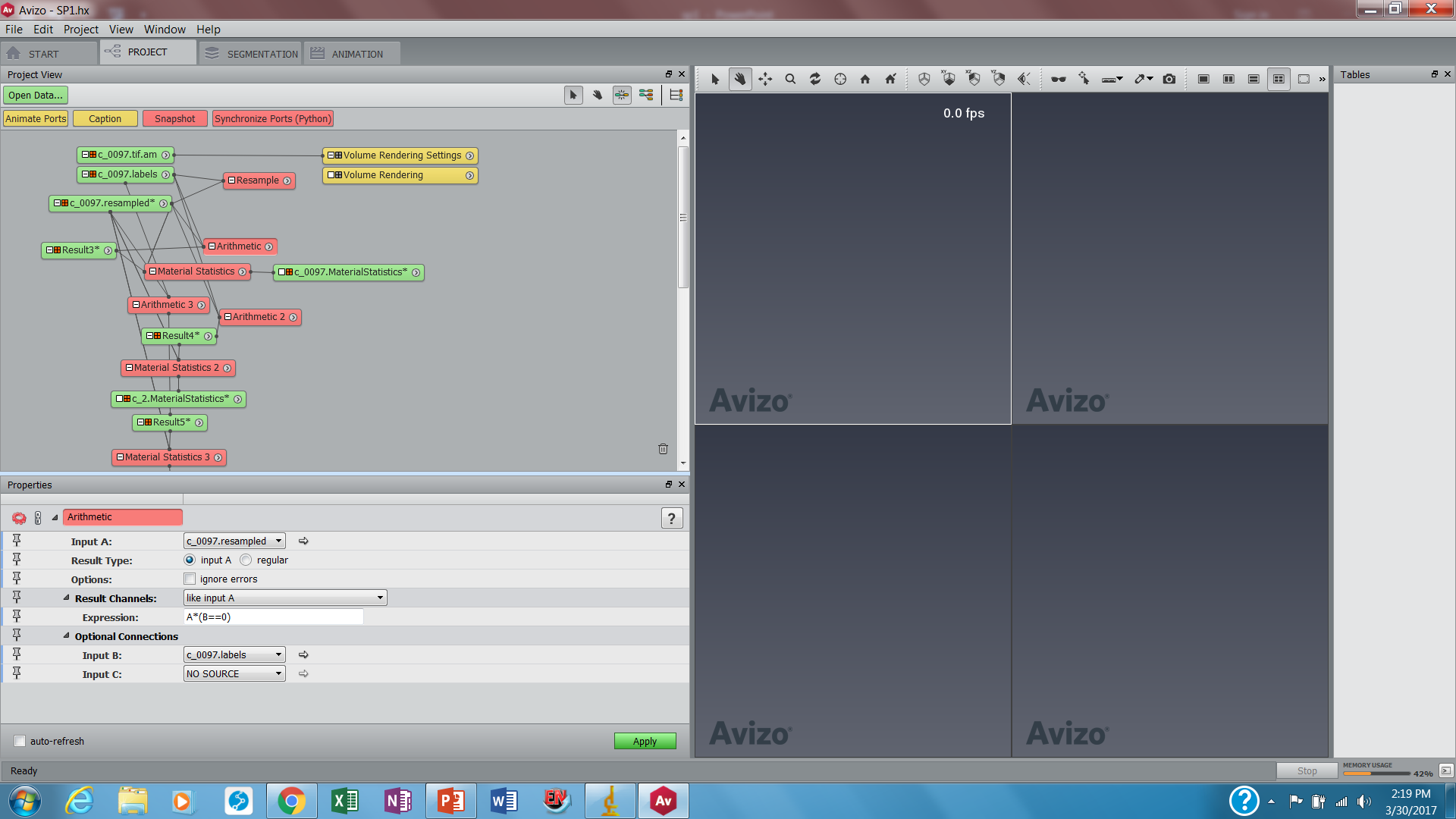
**

**Fig. S4.2** Screenshot of arithmetic module and its settings used in void volume and area calculation flowchart, for μ-CT images of the berries of *S. repens*, using Avizo software

**
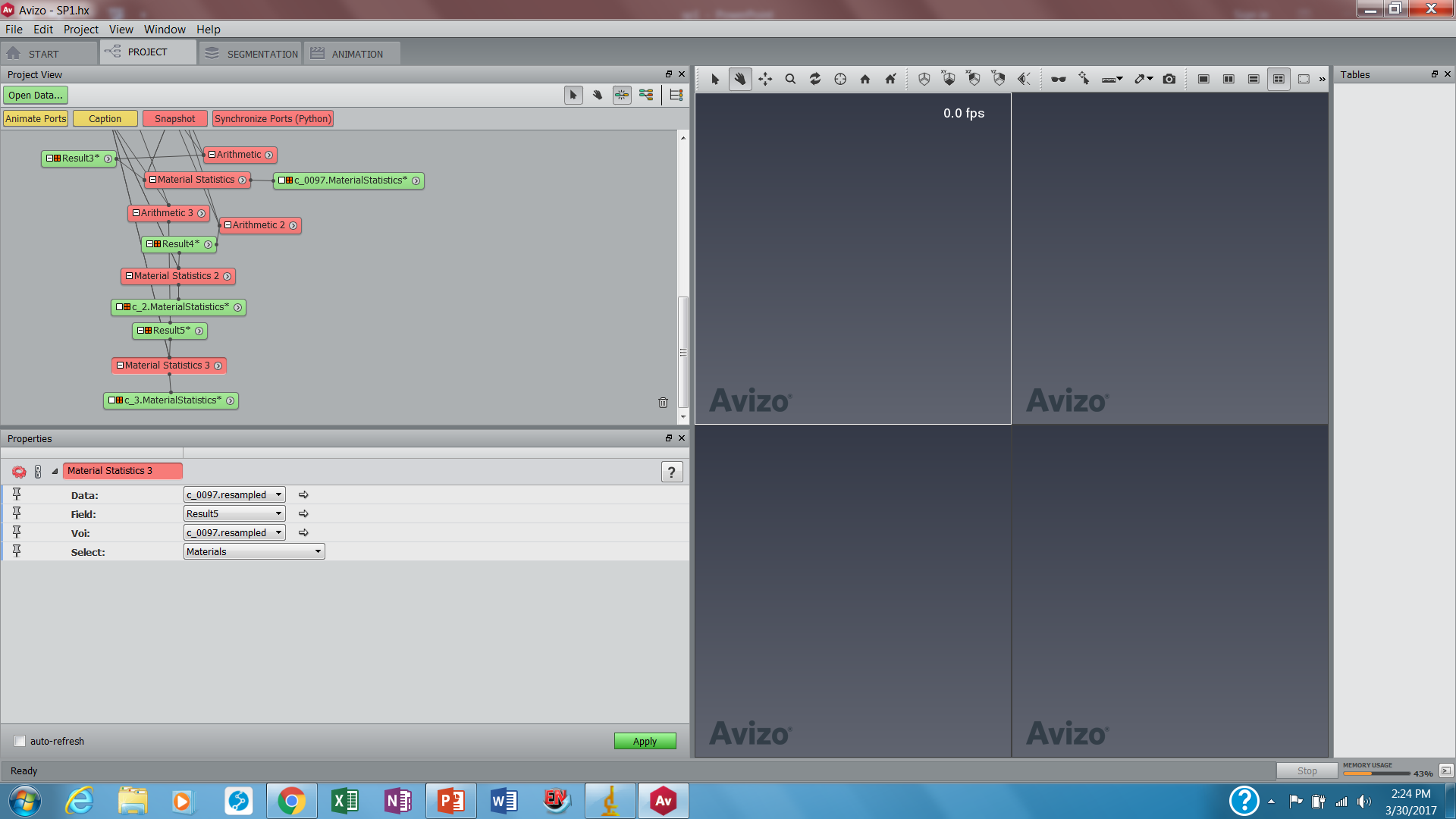
**

**S4.3** Screenshot of material statistics module used in void volume and area calculation flowchart, for μ-CT images of the berries of *S. repens*, using Avizo software

**S5 Image processing flowchart and settings for surface area calculation of μ-CT data, with Avizo software**

**
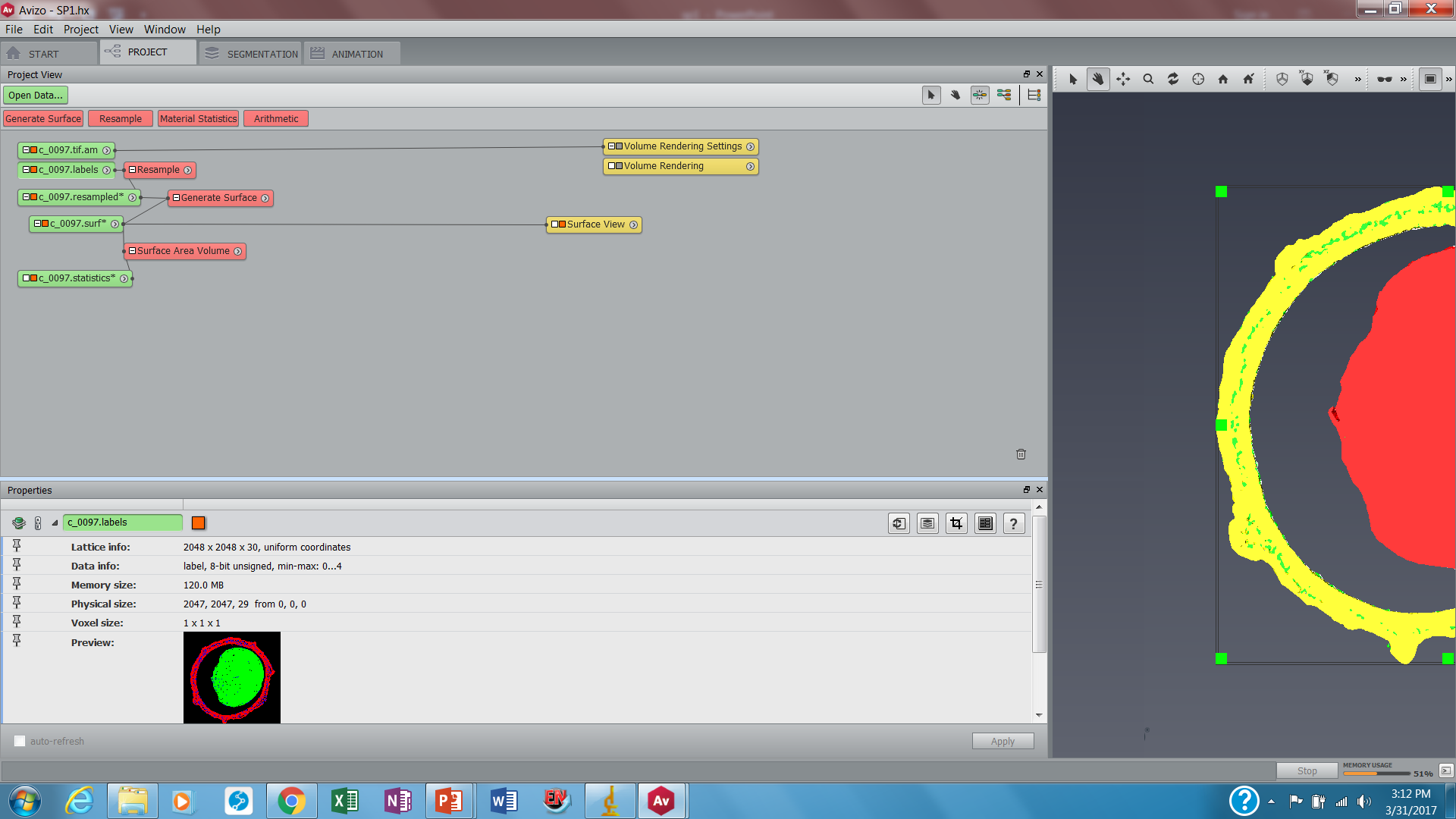
**

**Fig. S5.1** Screenshot of surface area calculation flowchart used for μ-CT images of the berries of *S. repens*, using Avizo software

**
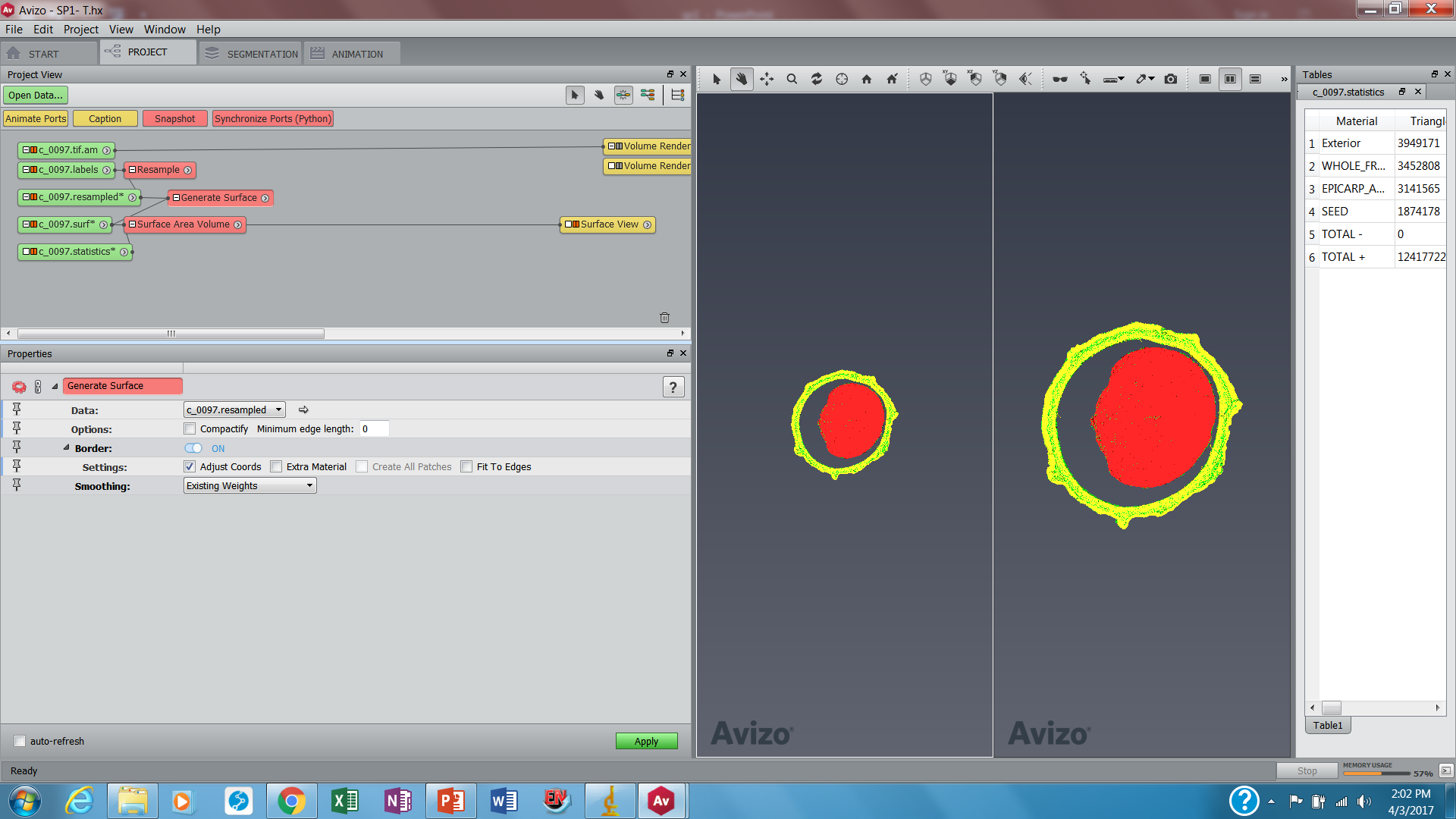
**

**S5.2** Screenshot of generate surface module used in surface area calculation flowchart, for μ-CT images of the berries of *S. repens*, using Avizo software

**
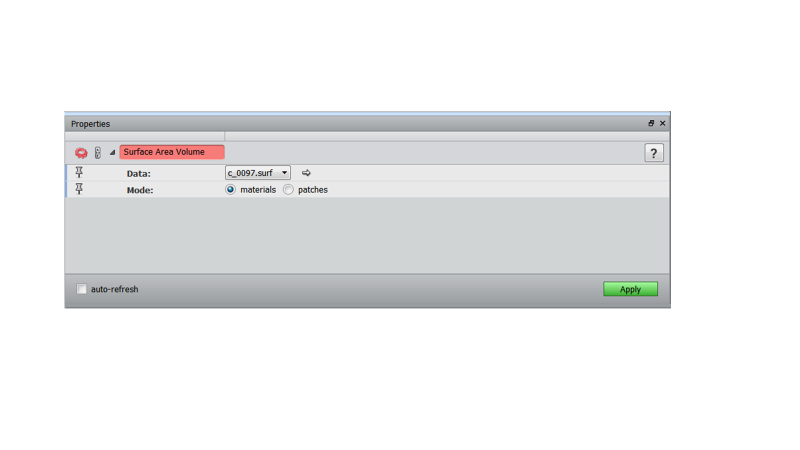
**

**S5.3** Screenshot of surface area volume module used in surface area calculation flowchart, for μ-CT images of the berries of *S. repens*, using Avizo software

**S6 Image processing flowchart and settings for porosity calculation of μ-CT data, with Avizo software**

**
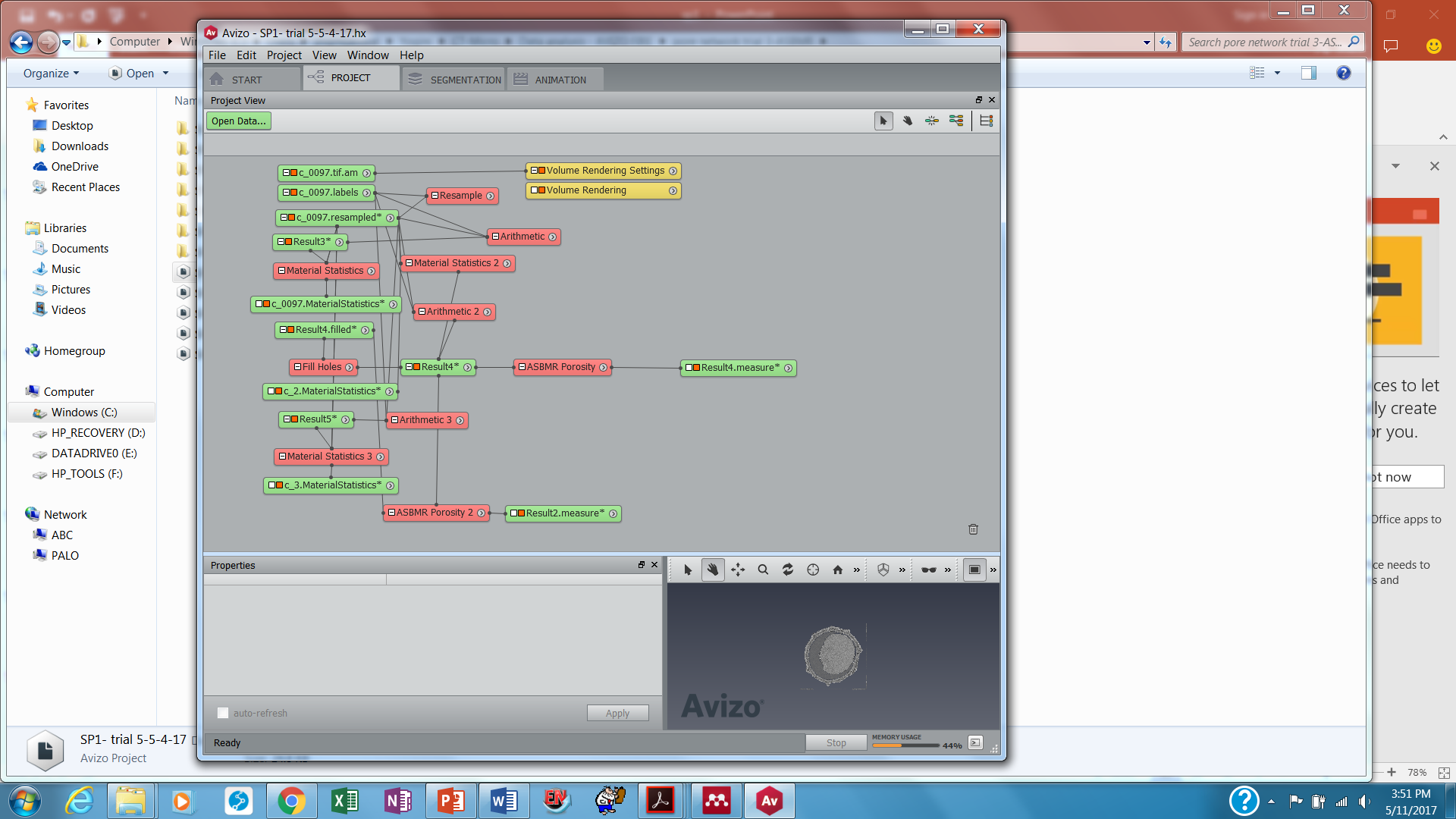
**

**Fig. S6.1** Screenshot of porosity calculation flowchart used for μ-CT images of the berries of *S. repens*, using Avizo software

**
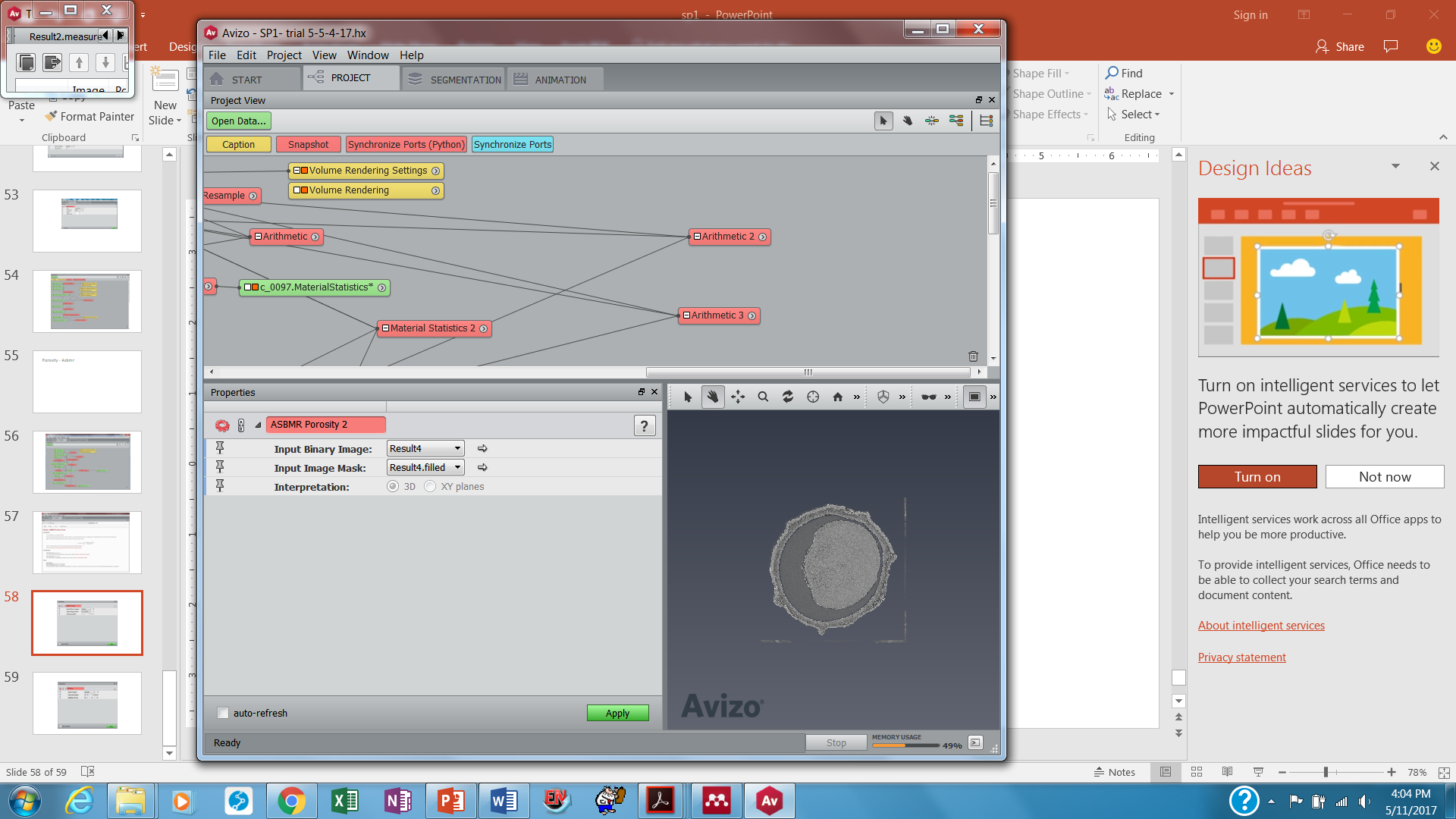
**

**Fig. S6.2** Screenshot of ASBMR porosity module, used in porosity estimation for μ-CT images of the berries of *S. repens*, using Avizo software

**S7 Image processing flowchart and settings for calculation of tortuosity related parameters for μ-CT data, with Avizo software**

**
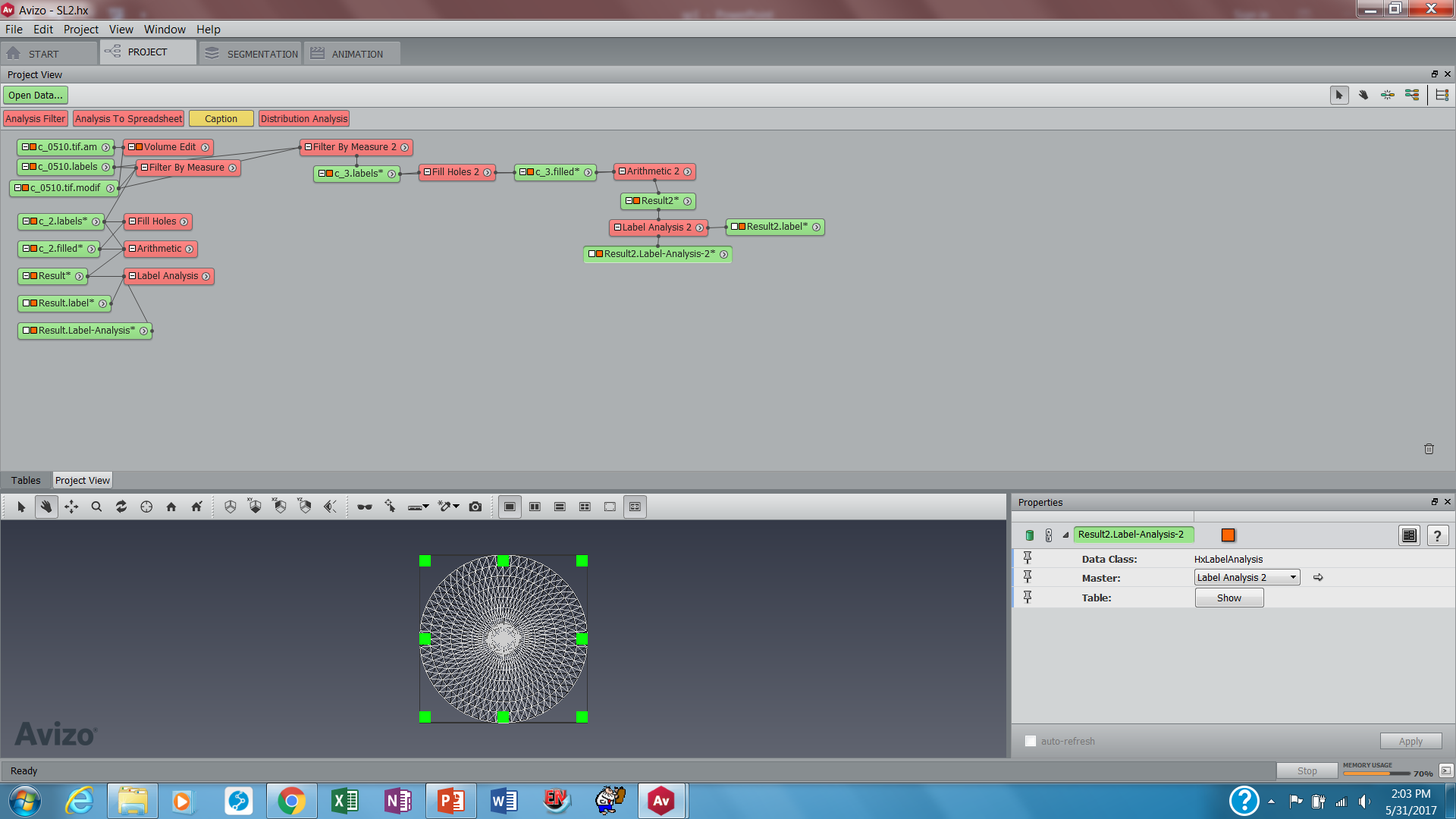
**

**Fig. S7.1** Screenshot of flowchart for calculation of tortuosity related parameters for μ-CT images of the berries of *S. repens*, using Avizo software

**
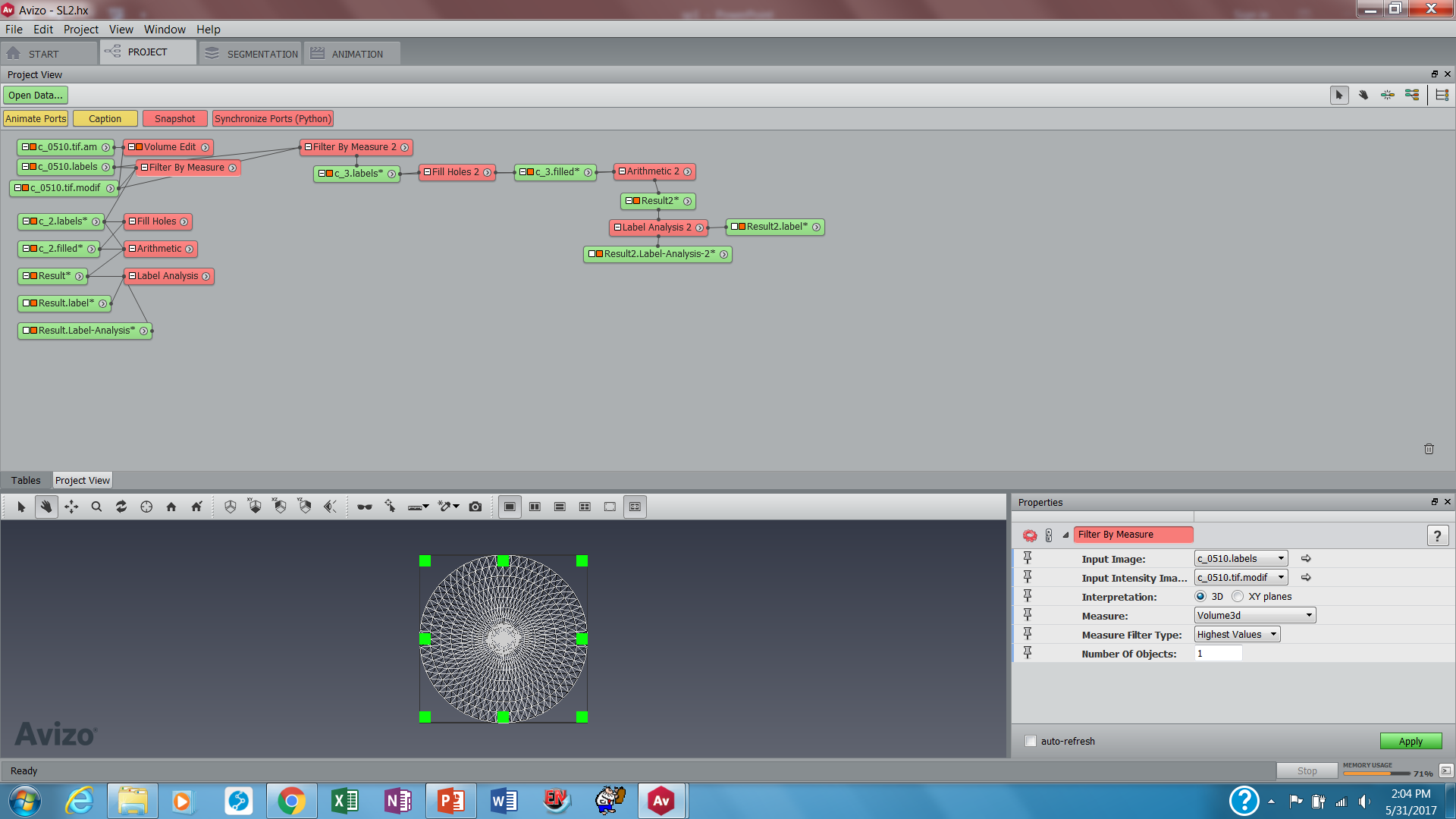
**

**Fig. S7.2** Screenshot of filter by measure module, used in tortuosity estimation for μ-CT images of the berries of *S. repens*, using Avizo software

**
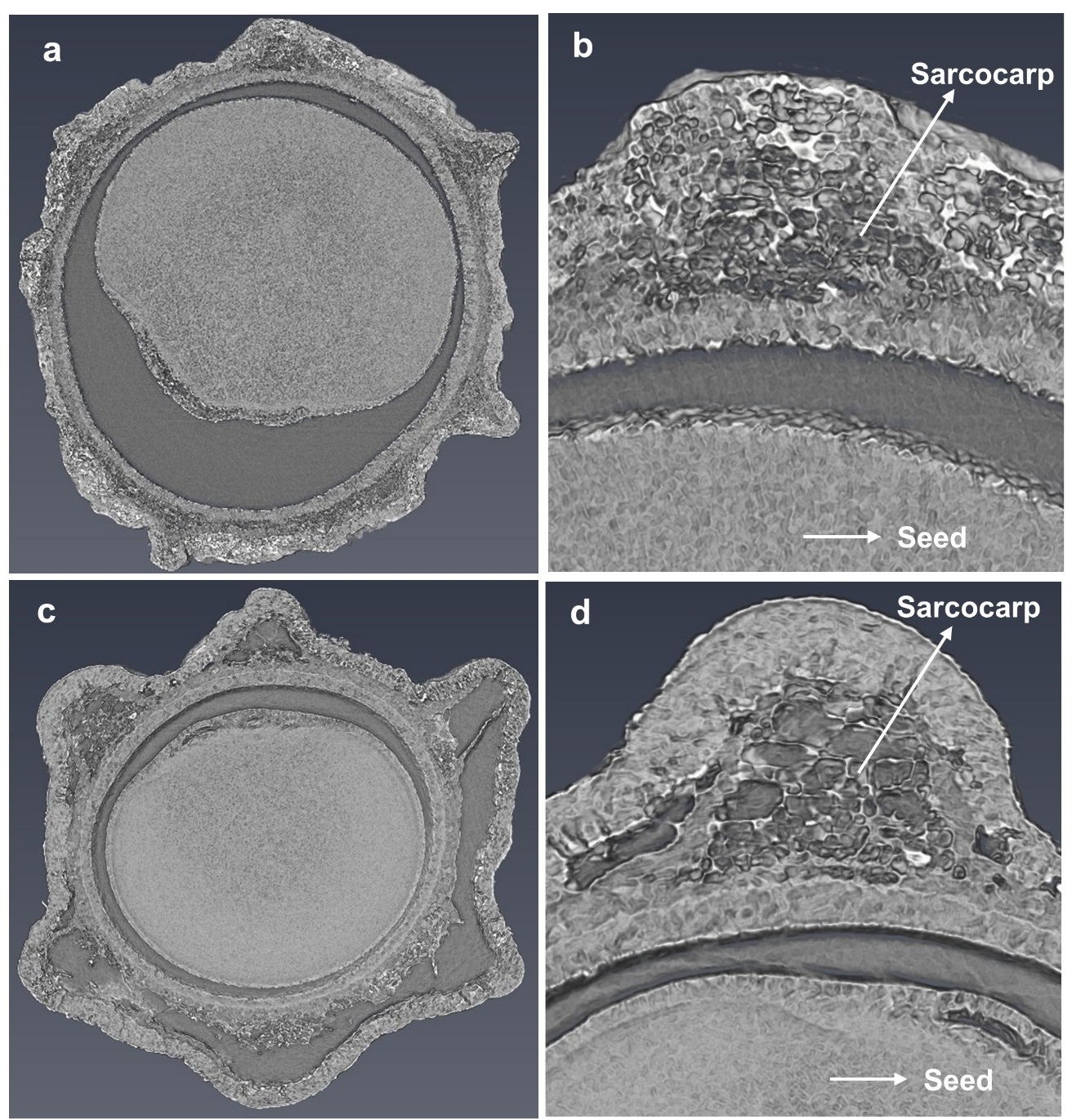
**

**Fig. S8 Porosity visualisation of berries of *S.repens* with Avizo software**

**a** and **b** represent the whole berry and a close view of a section of the 3D image respectively, for wild green variety of *S.repens*. **c** and **d** represent the whole berry and a close view of a section of the 3D image respectively, for silver variety of *S.repens*. The sarcocarp and mesocarp indicate porous network, whereas the seed indicates dense tissues.


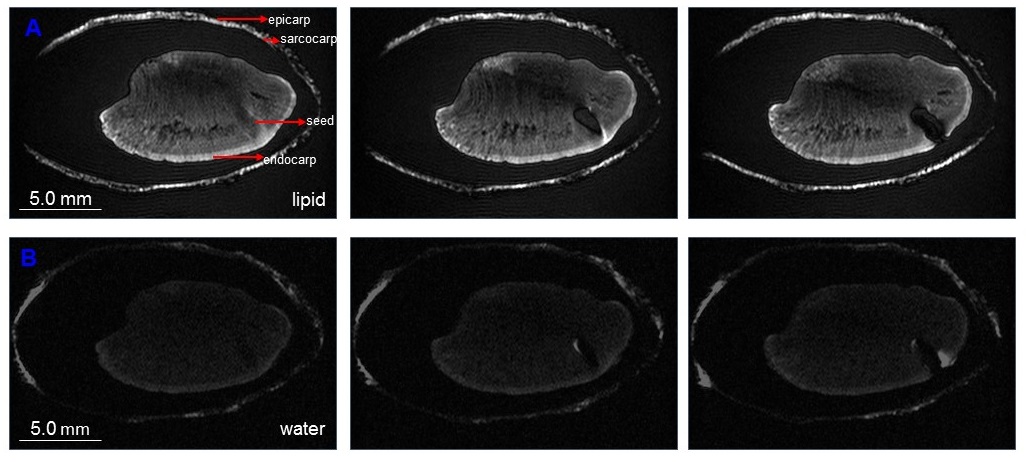


**Fig. S9 MRI images of longitudinal slices through a *S. repens* berry of wild variety (SP).** **A** and **B**, Represent the lipid and water distribution, respectively. For better visualization of the lower intensities, a non-linear grey-scale was used which overemphasizes the lower values and chops off the highest values by setting them to the maximum value of the grey-scale. Due to the distortion of the magnetic field at the left tip of the outer parts of the berry shell (left hand side in the image), the lipid signal is shifted to the water frequency and therefore appears in the water image.


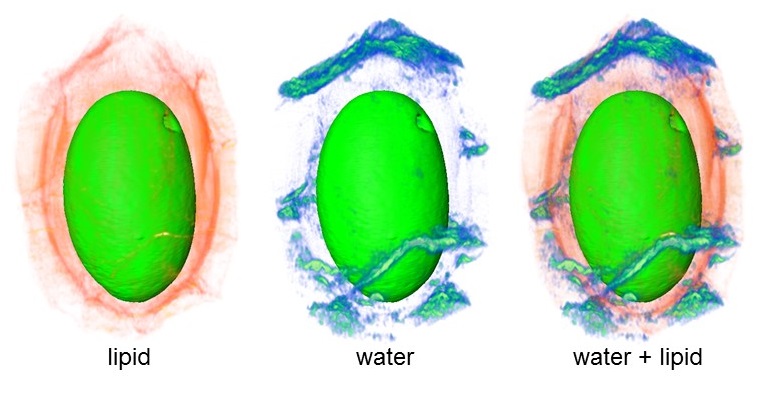


**Fig. S10** **Volume rendering of 3D datasets obtained by MRI analysis of berry of the silver variety (SL) of *S. repens*.** The lipid, water and overlay of lipid and water distribution signals are indicated in the left, middle and right 3D images. The dent in the seed's surface represents the location of the embryo.


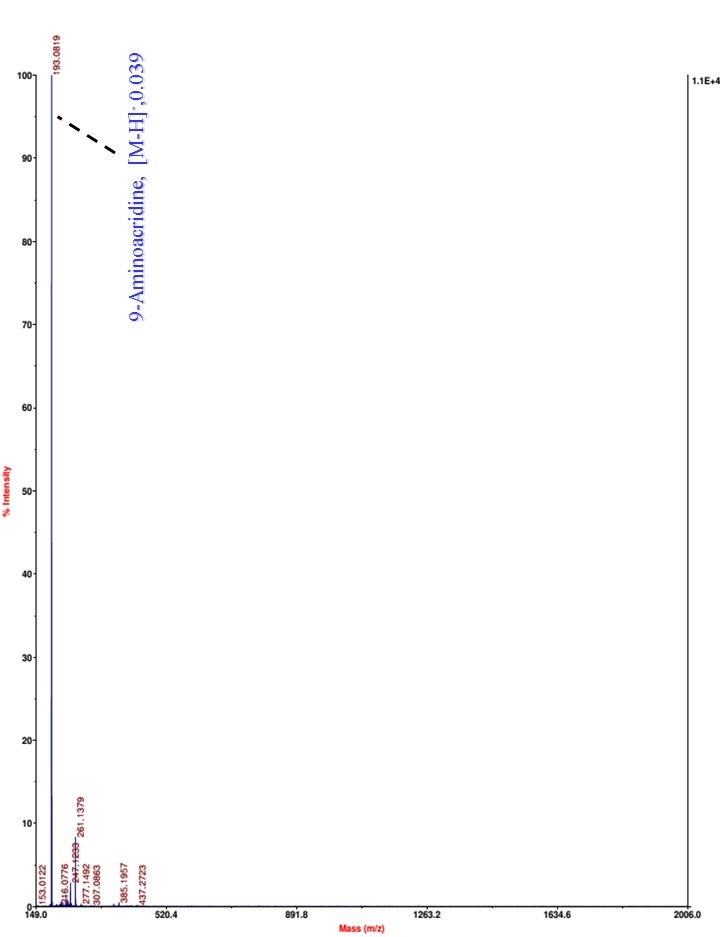


**Fig.S11** 9-Amino acridine used as matrix for analysis of samples in negative mode by MALDI-TOF/TOF technique.


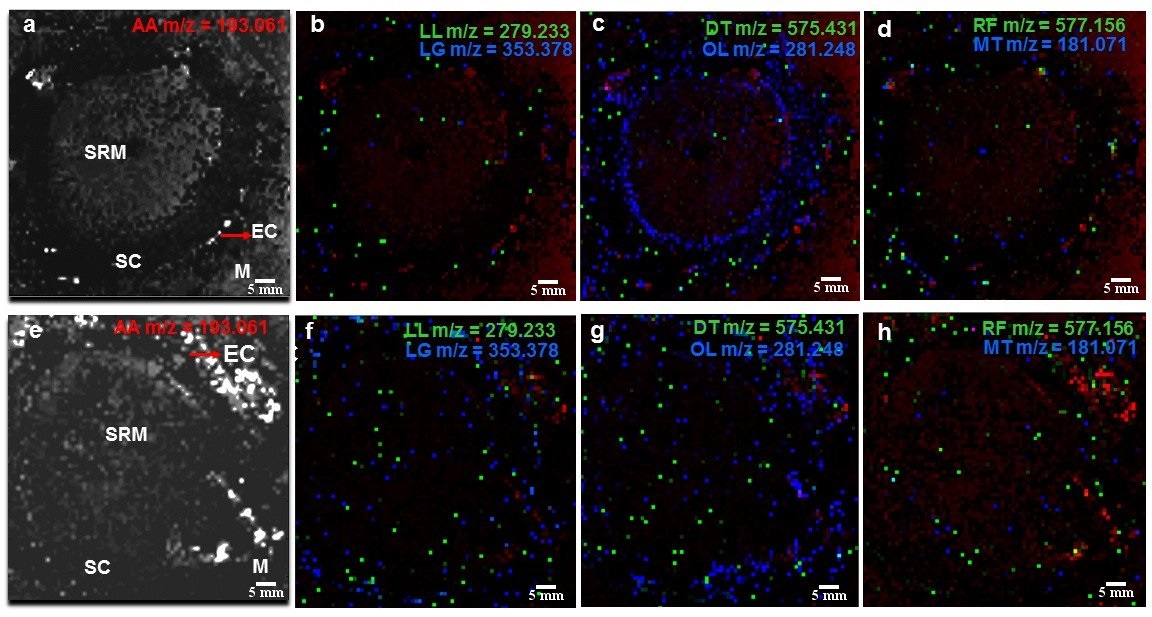


**Fig.** **S12 MALDI-MSI of representative metabolite distribution in transverse sections of berries of *S. repens***

**a-d,** represent SP variety and **e-h,** represent SL variety with 9-Amino acridine (AA) (red, *m/z* 193.061) as the matrix. **a** and **e,** represent binary images of sections of SP and SL, respectively. **b,** and **f**, depict overlay of ion images for linoleic acid (green, *m/z* 279.233) and lignoceryl alcohol (blue, *m/z* 353.378). **c** and **g,** depict overlay of ion images for daucosterol (green, *m/z* 575.434) and 9-octadecenoic acid (blue, *m/z* 281.248). **d** and **h,** depict overlay of ion images for rhoifolin (green, *m/z* 577.156) and mannitol (blue, *m/z* 181.071). All images were generated using TIC normalization method with a *m/z* window of 50 ppm. **EC** represents the epicarp, **SC** the sarcocarp, **SRM** is the region of seed location deposited by matrix and **M** represents the deposited matrix.

**
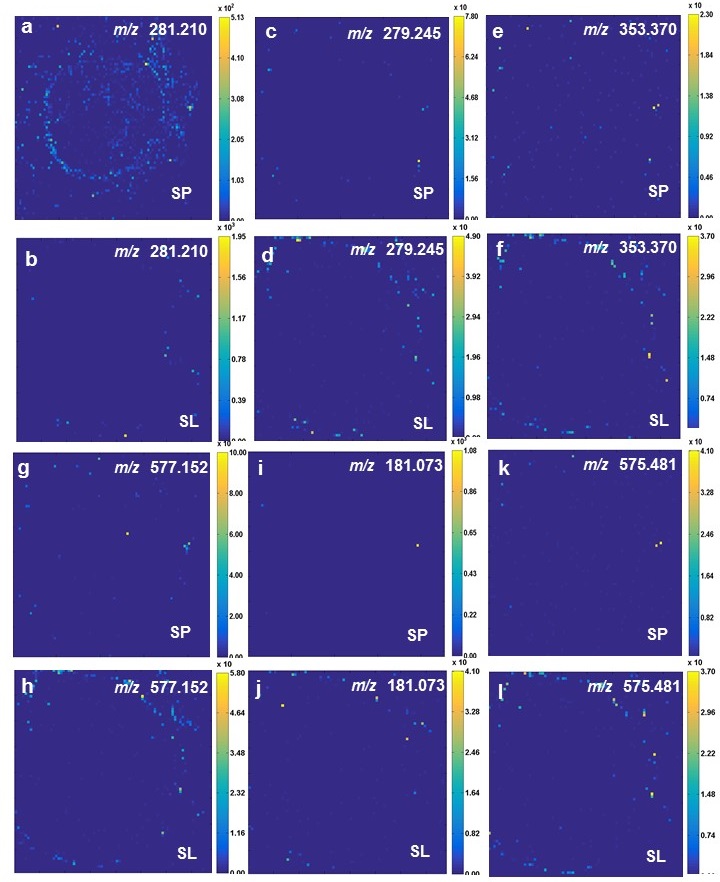
**

**Fig. S13** **MALDI-MSI images of selected metabolite ions in sections of *S. repens* berries**

The metabolites identified based on their *m/z* value in METLIN database and literature are 9-octadecenoic acid (*m/z* 281.244), linoleic acid (*m/z* 279.245), lignoceryl alcohol (*m/z* 353.373), daucosterol (*m/z* 575.481, rhoifolin (*m/z* 577.152) and mannitol (blue, *m/z* 181.073). SP and SL denote sections of wild green and silver varieties of *S. repens*. Each image represents [M-H]^-^ ions of selected metabolite generated with *m/z* window of 50 ppm.

**
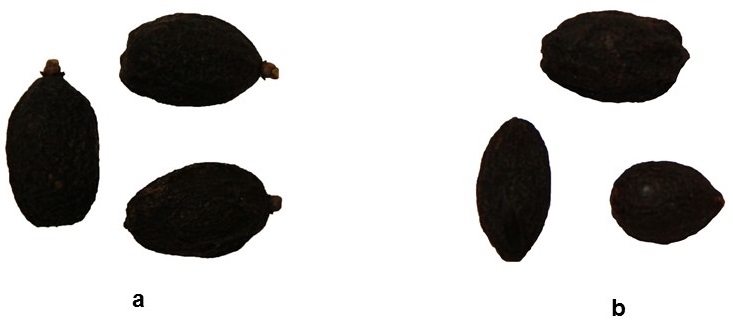
**

**Fig. S14 Pictorial representation of whole berries of *S. repens.***

**a**, represents the silver variety and **b**, represents the wild green variety of berries.

**
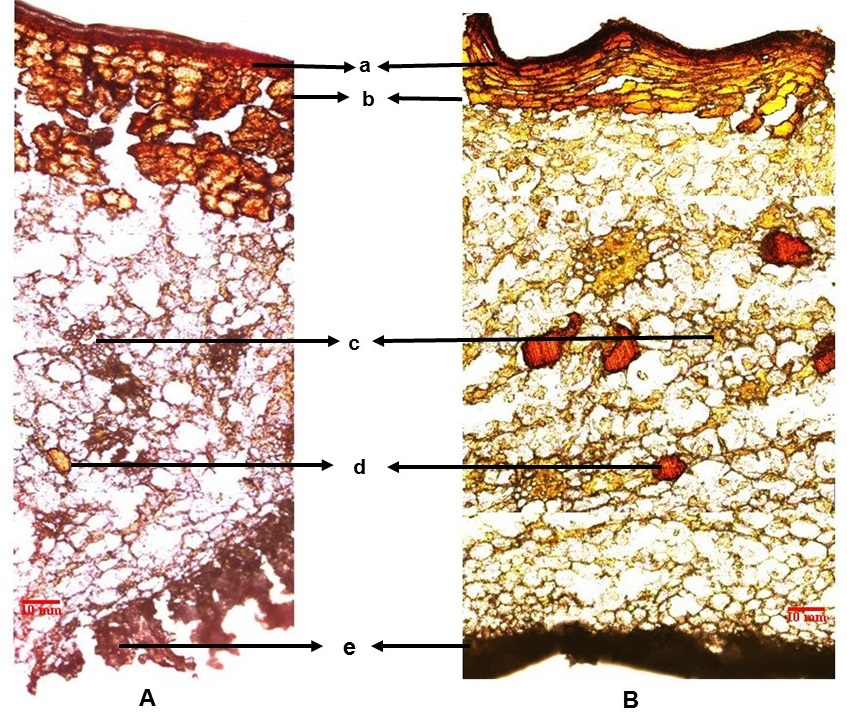
**

**Fig. S15 Microscopic features of whole berries of *S. repens*.**

A Zeiss Palm Microbeam system microscope was used at 5X magnification with 10 mm scale bar. **A**, silver and **B,** wild green variety tissue sections of *S. repens* berries. Tissues are represented by **a,** epidermis **b,** oil containing cells of the epicarp, **c,** sclerotic cells of sarcocarp, **d,** oil containing cells of sarcocarp, and **e,** endocarp.

**
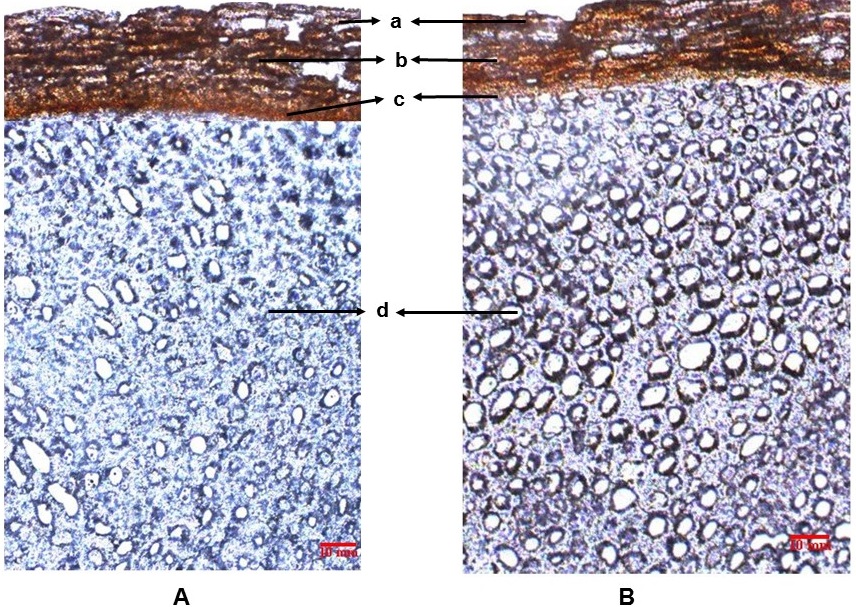
**

**Fig. S16 Microscopic features of seeds of *S. repens***

A Zeiss Palm Microbeam system microscope was used at 5X magnification with 10 mm scale bar. **A**, silver variety and **B**, wild green variety of *S. repens* variety. Tissues are represented by **a**, epidermis, **b**, parenchyma cells **c**, perisperm and **d**, endosperm.

**
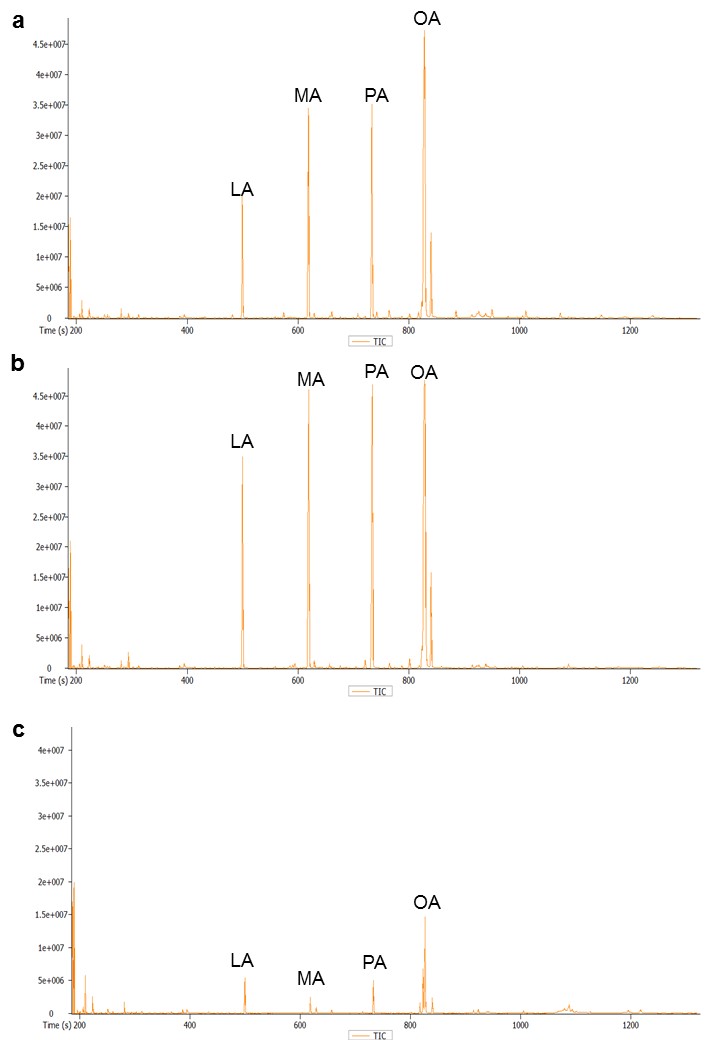
**

**Fig. S17** **GC-MS chromatograms of laser dissected tissues of *S. repens* (wild green variety) berries**.

**a**, epicarp, **b**, sarcocarp and **c**, seed of berries. LA, MA, PA and OA represent dodecanoic acid, myristic acid, palmitic acid and 9-octadecenoic acid, respectively.

**
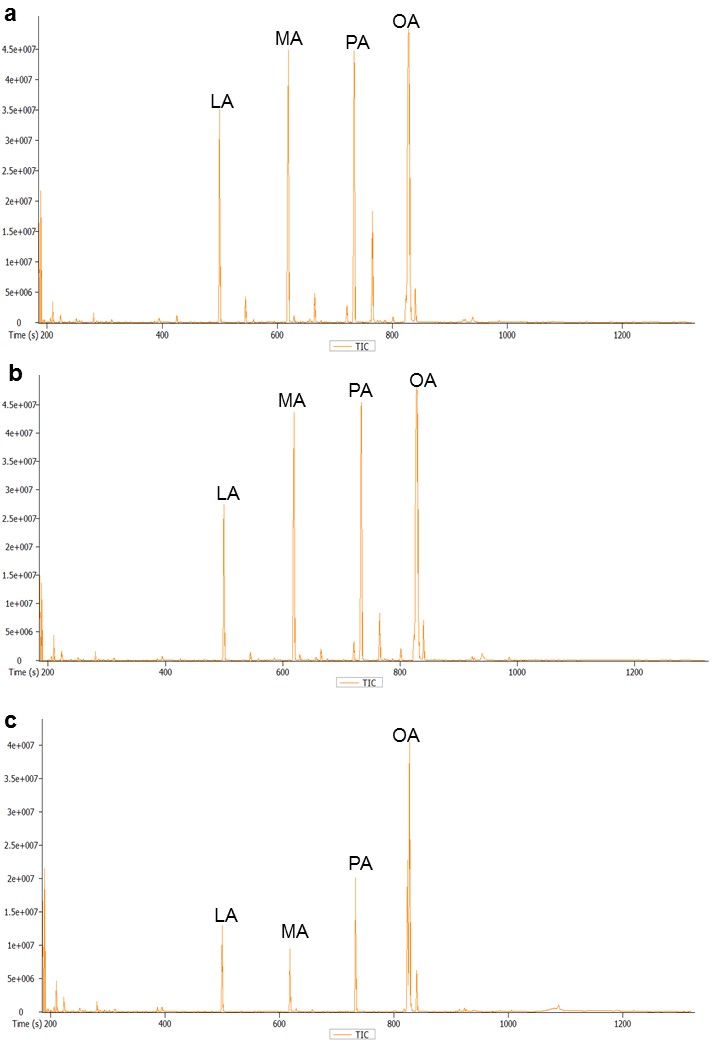
**

**Fig. S18** **GC-MS chromatograms of laser dissected tissues of *S. repens* (silver variety) berries**.

**a**, epicarp, **b**, sarcocarp and **c**, seed of berries. LA, MA, PA and OA represent dodecanoic acid, myristic acid, palmitic acid and 9-octadecenoic acid, respectively.

**
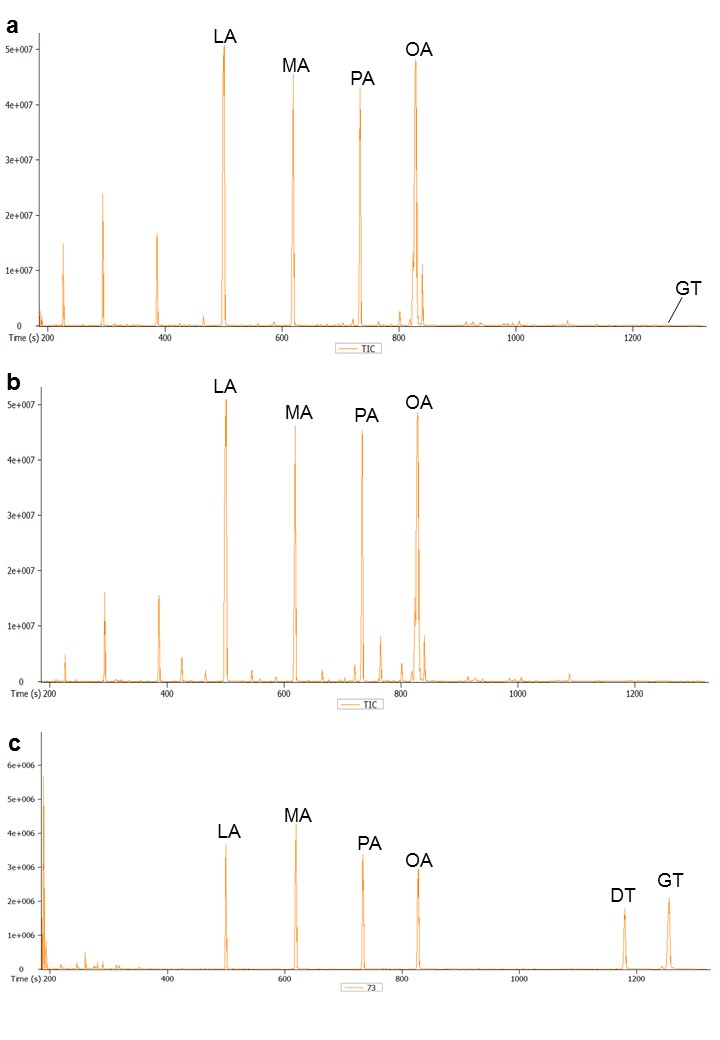
**

**Fig. S19 GC-MS chromatograms of extracts of whole berries of *Serenoa repens*.**

**a**, extract of whole fruit of *S. repens* (wild green variety), **b**, extract of whole fruit of *S. repens* (silver variety) and, **c**, standard compounds. LA, MA, PA, OA, DT and GT represent dodecanoic acid, myristic acid, palmitic acid, 9-octadecenoic acid, delta tocopherol and gamma tocopherol, respectively.

**S20 Metabolites identified by GC-MS analysis for berries of *S. repens*.**

| **Putative Compound Name** | **Formula** | **Theoretical Molecular Weight** | **Precursor m/z** | **Retention Time** | **RI (iu)** | **SP** | **SL** | **SP–LCM tissues** | | | **SL – LCM tissues** | | |
| --- | --- | --- | --- | --- | --- | --- | --- | --- | --- | --- | --- | --- | --- |
|  |  |  |  |  |  |  |  | **E** | **S** | **D** | **E** | **S** | **D** |
| **Fatty acids** | | | | | | | | | | | | | |
| Palmitic acid | C_16_H_32_O_2_ | 256.430 | 132 | 734.444 | 1987 | **+** | **+** | **+** | **+** | **+** | **+** | **+** | **+** |
| 9-octadecenoic acid | C_18_H_34_O_2_ | 282.468 | 84 | 828.316 | 2194 | **+** | **+** | **+** | **+** | **+** | **+** | **+** | **+** |
| Stearic acid | C_18_H_36_O_2_ | 284.484 | 132 | 840.429 | 2186 | **+** | **+** | **+** | **+** | **+** | **+** | **+** | **+** |
| Arachidic acid | C_20_H_40_O_2_ | 312.538 | 132 | 938.465 | 2385 | **+** | **+** | **+** | **+** | **+** | **+** | **+** | **+** |
| Dodecanoic acid | C_12_H_24_O_2_ | 200.322 | 117 | 499.762 | 1590 | **+** | **+** | **+** | **+** | **+** | **+** | **+** | **+** |
| Palmitoleic acid | C_16_H_30_O_2_ | 254.414 | 117 | 720.814 | 1995 | **+** | **+** | **+** | **+** | **-** | **+** | **+** | **+** |
| Myristic acid | C_14_H_28_O_2_ | 228.376 | 285 | 619.753 | 1788 | **+** | **+** | **+** | **+** | **+** | **+** | **+** | **+** |
| Pentadecanoic acid | C_15_H_30_O_2_ | 242.403 | 74 | 665.175 | 1814 | **+** | **+** | **+** | **+** | **+** | **+** | **+** | **+** |
| Palmitelaidic acid | C_16_H_30_O_2_ | 254.414 | 117 | 721.195 | 1995 | **+** | **+** | **+** | **+** | **-** | **+** | **+** | **+** |
| cis-6-Octadecenoic acid | C_18_H_34_O_2_ | 282.468 | 74 | 765.104 | 2085 | **+** | **+** | **+** | **+** | **-** | **+** | **+** | **-** |
| cis-10-Heptadecenoic acid | C_17_H_32_O_2_ | 268.441 | 75 | 774.188 | 2095 | **+** | **+** | **+** | **+** | **+** | **+** | **+** | **+** |
| Linoelaidic acid | C_18_H_32_O_2_ | 280.452 | 337 | 823.774 | 2202 | **+** | **+** | **+** | **+** | **+** | **+** | **+** | **+** |
| Gondoic acid | C_20_H_38_O_2_ | 310.522 | 367 | 926.731 | 2393 | **+** | **+** | **+** | **+** | **+** | **+** | **+** | **+** |
| 1-Monooleoylglycerol | C_21_H_40_O_4_ | 356.547 | 103 | 1087.98 | 2788 | **+** | **+** | **+** | **+** | **+** | **-** | **-** | **+** |
| Lignoceric acid | C_24_H_48_O_2_ | 368.646 | 132 | 1137.19 | 2782 | **+** | **+** | **+** | **+** | **+** | **+** | **+** | **+** |
| Pentacosanoic acid | C_25_H_50_O_2_ | 382.673 | 132 | 1205.7 | 2882 | **+** | **+** | **-** | **+** | **-** | **+** | **+** | **+** |
| Methyl tetradecanoate | C_15_H_30_O_2_ | 242.403 | 74 | 544.806 | 1680 | **+** | **+** | **-** | **+** | **-** | **+** | **+** | **-** |
| **Phenols** | | | | | | | | | | | | | |
| Gamma-Tocopherol | C_28_H_48_O_2_ | 416.690 | 488 | 1252.26 | 3113 | **+** | **+** | **-** | **+** | **-** | **+** | **+** | **-** |
| **Other compounds** | | | | | | | | | | | | | |
| Squalene | C_30_H_50_ | 410.730 | 81 | 1125.45 | 2914 | **+** | **+** | **-** | **+** | **+** | **+** | **-** | **+** |

**SPW** and **SLW** denote the extracts of whole berries of the wild green and silver varieties, respectively. **E**, **S,** and **D** denote laser micro-dissected epicarp, sarcocarp and seed tissues, respectively. **RI** denotes the Kovat’s retention indices for each identified metabolite. (-) indicates the absence of the constituent in the listed sample group.

**S21 Concentrations of fatty acids in different varieties of berries of *S. repens,* identified by GC-MS analysis.**

| **Fatty acids** | **SL (mg/g)** | **SP (mg/g)** |
| --- | --- | --- |
| Dodecanoic acid (lauric acid) | 32.048 ± 24.556 | 28.247 ± 18.229 |
| Myristic acid | 4.084 ± 3.326 | 5.101 ± 1.487 |
| Palmitic acid | 5.342 ± 3.610 | 5.111 ± 1.531 |
| 9-octadecenoic acid (oleic acid) | 10.314 ± 6.681 | 10.430 ± 3.176 |

**SL** and **SP** represent the silver variety and wild green variety of *S. repens.* The concentrations were calculated on a dry weight basis.


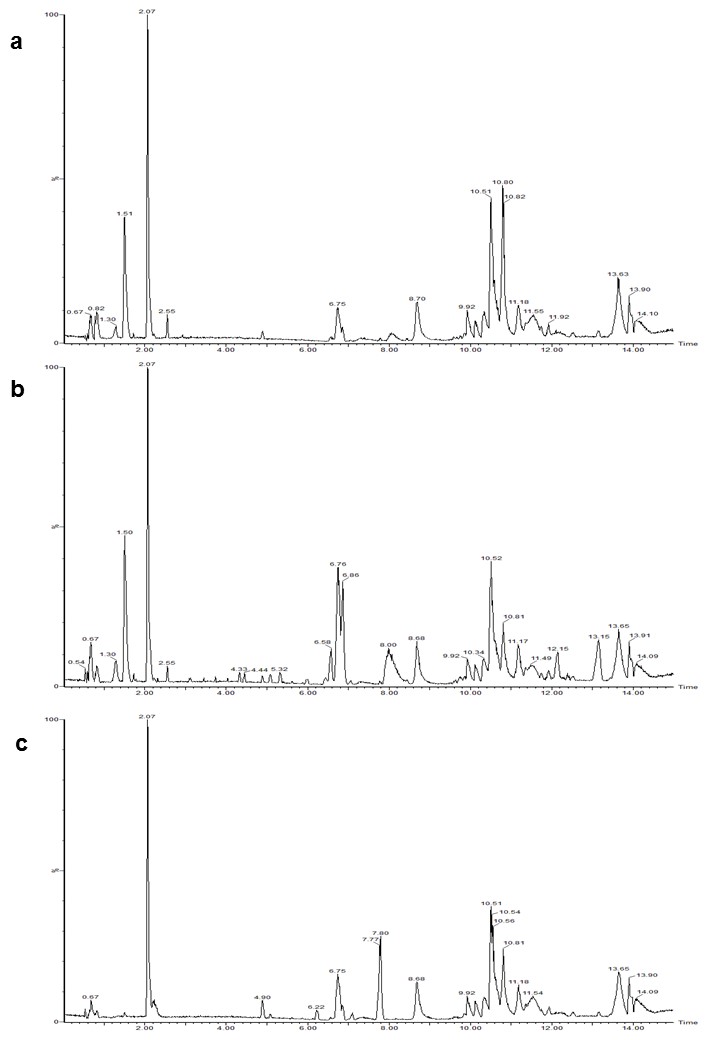


**Fig. S22 LC-MS base peak chromatograms of laser dissected tissues of *S. repens* (wild green variety) berries** **in positive mode.**

**a**, epicarp, **b**, sarcocarp **c**, seed tissues. p-chlorophenylalanine used as internal standard is eluted at R_t_ = 2.06 min.


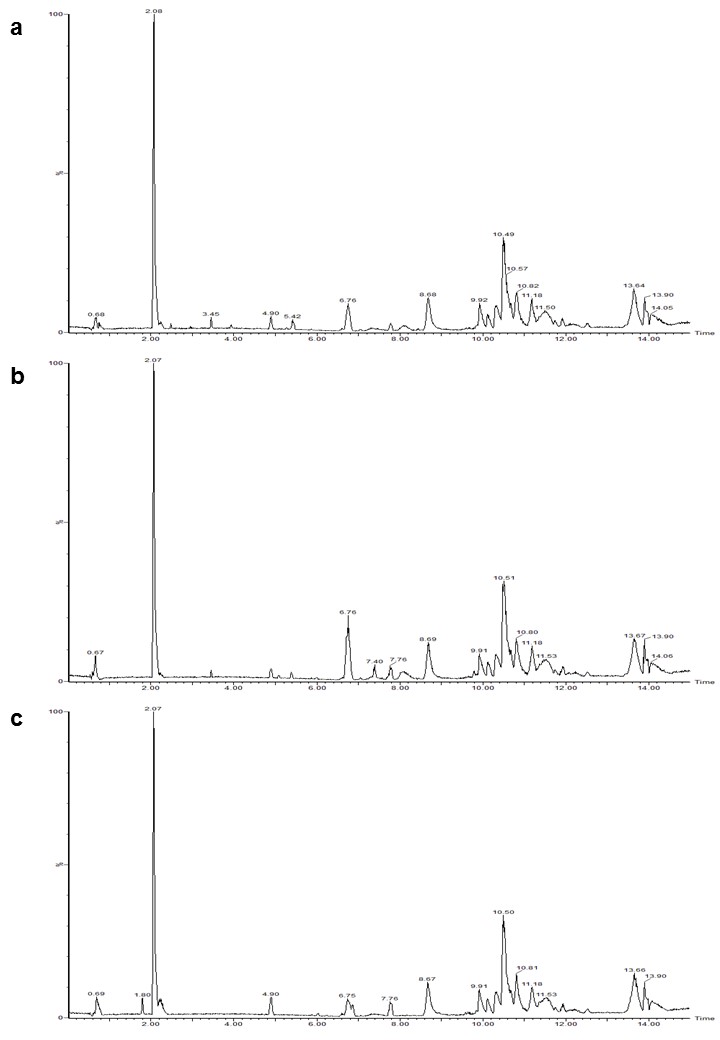


**Fig. S23 LC-MS base peak chromatograms of laser dissected tissues of *S. repens* (silver variety) berries in positive mode**

**a**, epicarp, **b**, sarcocarp **c**, seed tissues. p-chlorophenylalanine used as internal standard is eluted at R_t_ = 2.06 min.

**
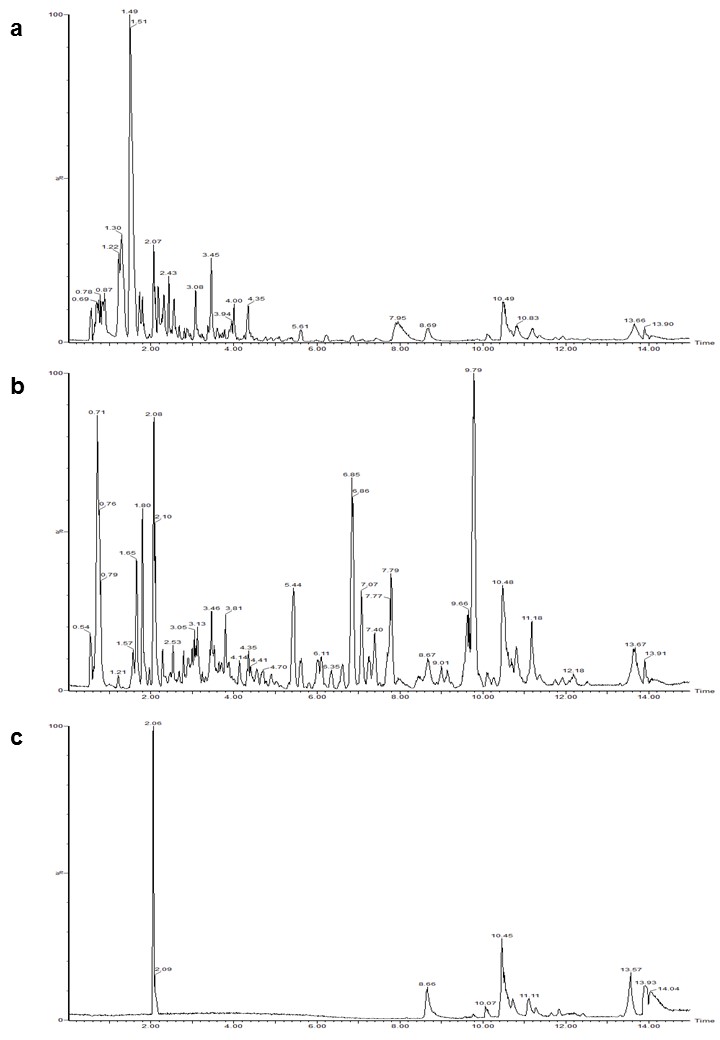
**

**Fig. S24 LC-MS base peak chromatograms of extracts of whole berries of *S. repens* analysed in positive mode.**

**a**, extract of whole berries of *S. repens* (wild green variety) **b**, extract of whole berries of *S. repens* (silver variety) and **c**, methanol used as blank spiked with internal standard (p-chlorophenylalanine).


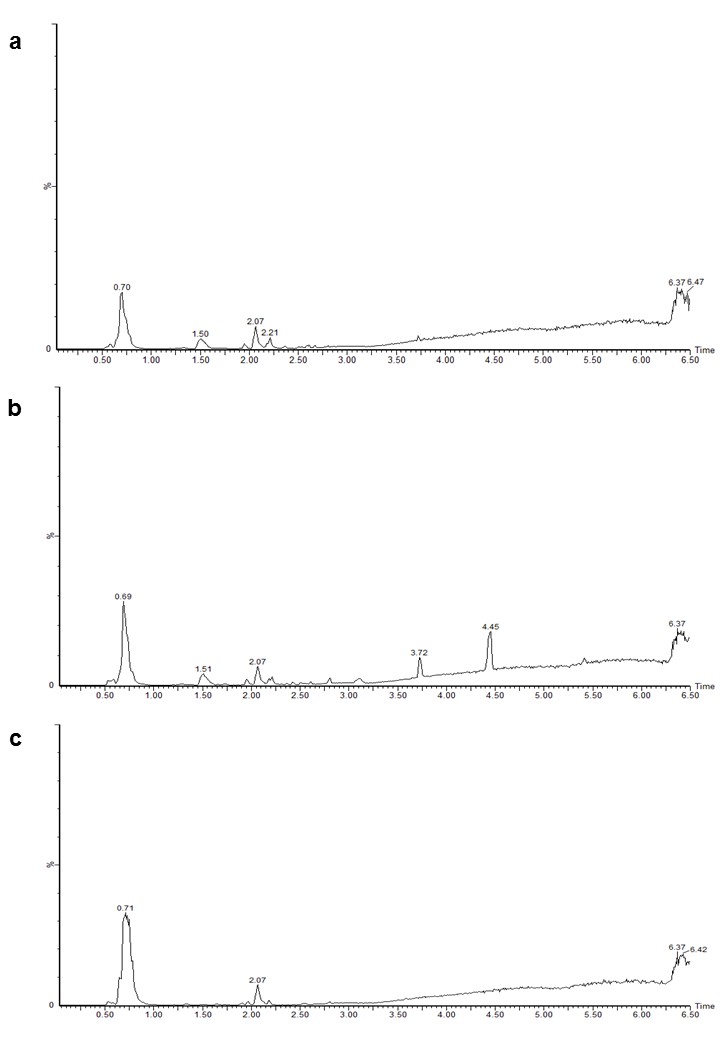


**Fig. S25** **LC-MS base peak chromatograms of laser dissected tissues of *S. repens* (wild green variety) berries in negative mode.**

**a**, epicarp **b**, sarcocarp and **c**, seed tissues. p-chlorophenylalanine used as internal standard is eluted at R_t_ = 2.06 min.

**
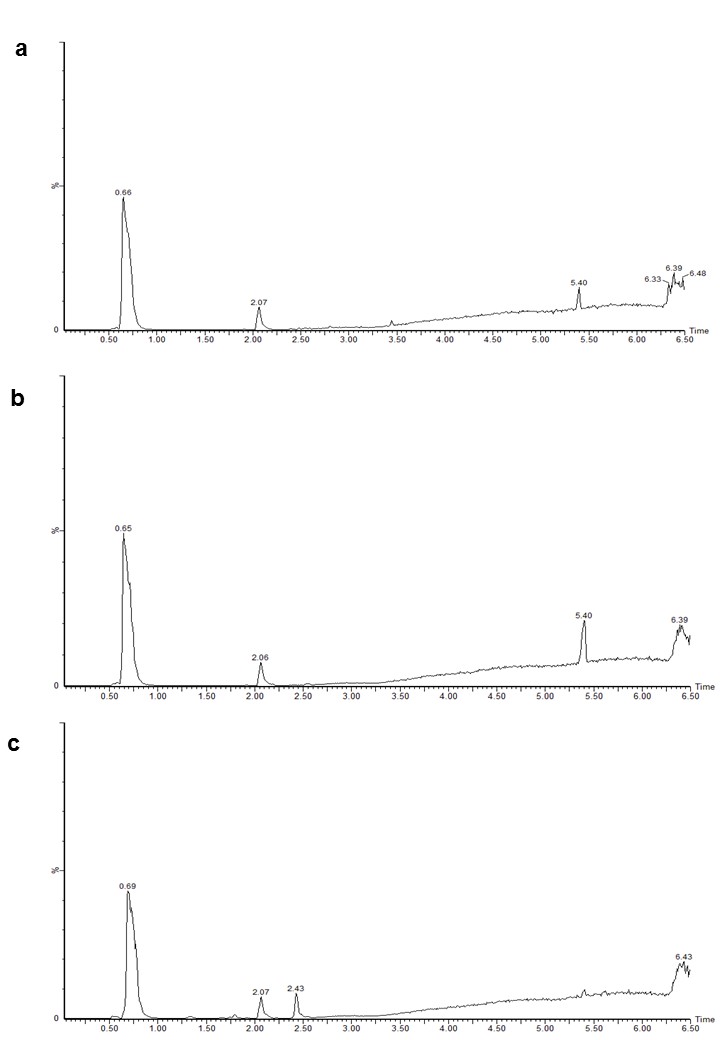
**

**Fig. S26** **LC-MS base peak chromatograms of laser dissected tissues of *S. repens* (silver variety) berries in negative mode**.

**a**, epicarp **b**, sarcocarp **c**, seed tissues. p-chlorophenylalanine used as internal standard is eluted at R_t_ = 2.06 min.


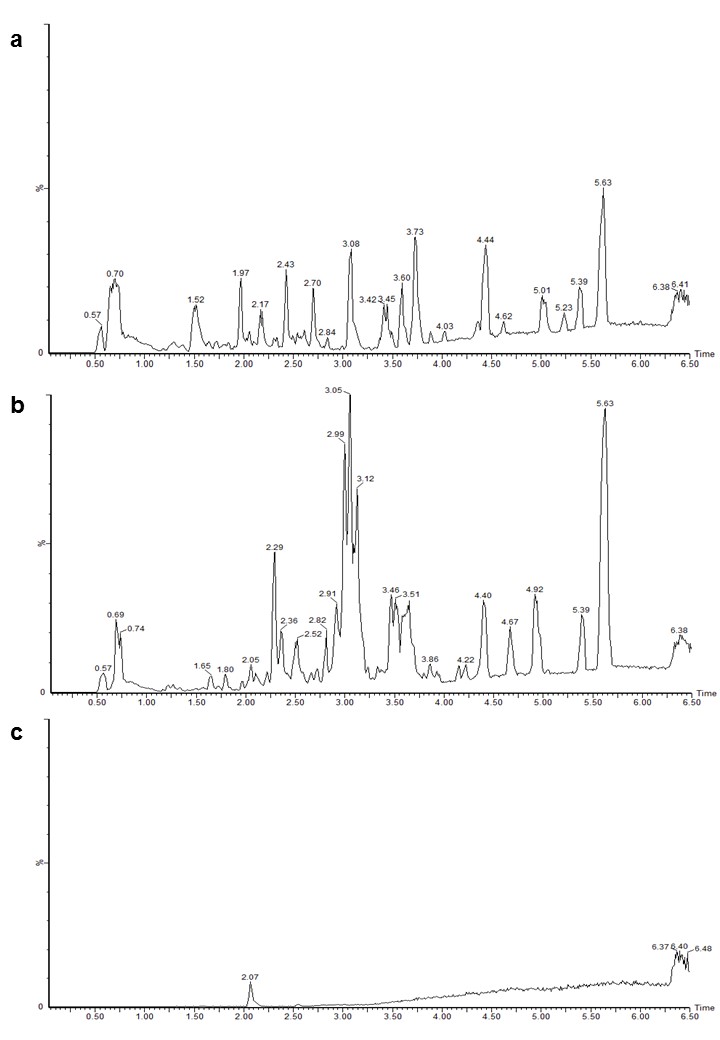


**Fig. S27 LC-MS base peak chromatograms of extracts of whole berries of *S. repens* analyzed in negative mode.**

**a**, extract of whole berries of *S. repens* (wild green variety) **b**, extract of whole berries of *S. repens* (silver variety) and **c**, methanol used as blank spiked with internal standard (p-chlorophenylalanine).

**S28: Metabolites identified by LC-MS analysis of berries of *S. repens*.**

| **Compound name** | **Formula** | **Theoretical molecular weight** | **Observed m/z** | **Adducts** | **Mass error (ppm)** | **Retention Time** | **SPW** | **SLW** | **SP–LCM tissues** | | | **SL – LCM tissues** | | |
| --- | --- | --- | --- | --- | --- | --- | --- | --- | --- | --- | --- | --- | --- | --- |
|  |  |  |  |  |  |  |  |  | **E** | **S** | **D** | **E** | **S** | **D** |
| **Flavonoids** | | | | | | | | | | | | | | |
| Kaempferol | C_15_H_10_O_6_ | 286.239 | 285.043 | M-H | 11.700 | 3.102 | + | + | + | + | + | + | + | + |
| Rhoifolin | C_27_H_30_O_14_ | 578.523 | 579.171 | M+H | 1.475 | 2.535 | + | + | - | - | - | - | - | - |
| Quercitrin | C_21_H_20_O_11_ | 448.380 | 447.096 | M-H | 6.403 | 2.571 | + | + | + | + | - | + | + | - |
| Rutin | C_27_H_30_O_16_ | 610.521 | 609.151 | M-H | 9.512 | 2.347 | + | + | - | - | - | - | - | - |
| Isoquercitrin | C_21_H_20_O_12_ | 464.379 | 463.093 | M-H | 12.440 | 2.428 | + | + | + | + | - | + | + | + |
| Apigenin 4'-glucoside | C_21_H_20_O_10_ | 432.381 | 433.118 | M+H | 11.934 | 2.401 | + | + | + | + | + | + | + | + |
| **Fatty acids** | | | | | | | | | | | | | | |
| Caproic acid | C_6_H_12_O_2_ | 116.160 | 115.078 | M-H | 13.204 | 2.544 | + | + | + | + | - | + | - | - |
| Myristic acid | C_14_H_28_O_2_ | 228.376 | 227.203 | M-H | 6.651 | 8.447 | + | + | + | + | + | + | + | + |
| 1-Monolaurin | C_15_H_30_O_4_ | 274.401 | 273.207 | M-H | -0.160 | 4.703 | + | + | - | + | - | - | + | - |
| Tridecanoic acid | C_13_H_26_O_2_ | 214.349 | 213.187 | M-H | 7.428 | 5.100 | + | + | + | + | + | + | + | + |
| Undecanoic acid | C_11_H_22_O_2_ | 186.295 | 187.115 | M+H | -19.070 | 4.146 | + | + | + | + | + | + | + | + |
| **Polyprenoids** | | | | | | | | | | | | | | |
| Geraniol | C_10_H_18_O | 154.253 | 153.129 | M-H | 9.170 | 3.427 | **+** | **+** | **-** | **+** | **+** | **+** | **+** | **+** |
| Farnesol | C_15_H_26_O | 222.372 | 245.189 | M+Na | 8.430 | 2.085 | **-** | **+** | **-** | **-** | **-** | **-** | **-** | **-** |
| **Saccharides** | | | | | | | | | | | | | | |
| Arabinose | C_5_H_10_O_5_ | 150.130 | 149.047 | M-H | 11.199 | 0.728 | + | + | + | + | + | + | + | + |
| Pectin | C_6_H_10_O_7_ | 194.139 | 193.037 | M-H | 11.514 | 0.665 | + | + | + | + | + | + | + | + |
| **Phytosterol** | | | | | | | | | | | | | | |
| Daucosterol | C_35_H_60_O_6_ | 576.859 | 577.440 | M+H | -9.374 | 13.827 | **+** | **+** | + | + | + | + | + | + |
| **Vitamins** | | | | | | | | | | | | | | |
| Ascorbic acid | C_6_H_8_O_6_ | 176.124 | 175.027 | M-H | 12.418 | 0.737 | + | + | + | + | + | + | + | + |
| Riboflavin | C_17_H_20_N_4_O_6_ | 376.369 | 377.147 | M+H | 4.898 | 2.103 | + | + | + | - | - | + | + | - |
| Niacin | C_6_H_5_NO_2_ | 123.111 | 124.040 | M+H | 9.961 | 1.474 | + | + | - | - | - | - | - | - |
| **Other organic compounds** | | | | | | | | | | | | | | |
| Ferulic acid | C_10_H_10_O_4_ | 194.186 | 193.052 | M-H | 10.144 | 3.111 | + | + | + | + | - | + | + | - |
| P-coumaric acid | C_9_H_8_O_3_ | 164.160 | 163.043 | M-H | 18.044 | 1.852 | + | + | + | + | + | + | + | + |

**SPW** and **SLW** denote the whole fruit extracts of the wild green and silver variety, respectively. **E**, **S** and **D** denote laser micro-dissected epicarp, sarcocarp and seed tissues, respectively. (-) indicates the absence of the constituent in the listed sample group.

**
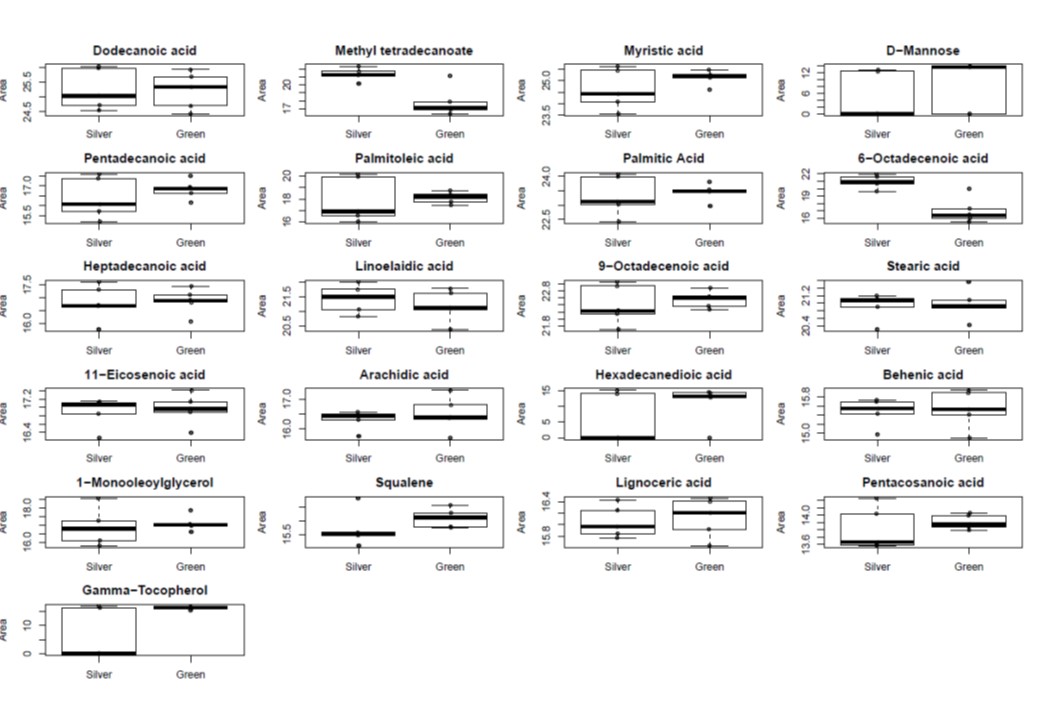
S29** Box and whiskers plots of the relative abundances of metabolites identified by GC-MS analysis.

**S30** Box and whiskers plots of the relative abundances of metabolites identified by LC-MS analysis. Significant metabolites are indicated with an asterisk (*).

**
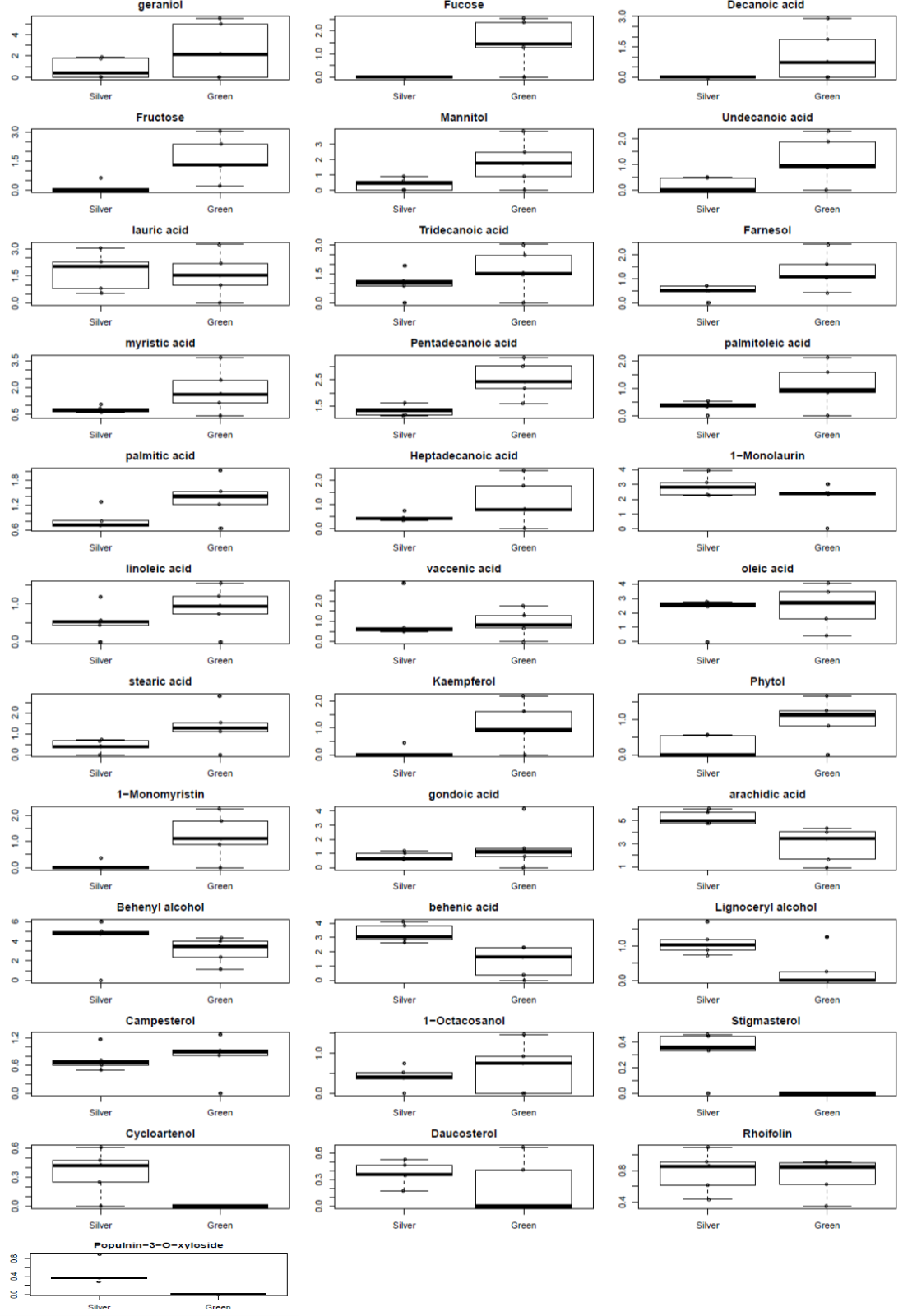
**

**S31** Box and whiskers plots of the relative abundances of selected metabolites identified by MALDI-TOF analysis.

**S32 Image processing of SR-μCT data.**

The 3D images were rendered from reconstruction of digital slices with VG Studio Max 3D construction software (version 2.1, Volume Graphics GmbH, Germany). Avizo Fire software 9.3.0 (FEI, Oregon, US) was used for the 3D tomography data analysis of the images. The image analysis of the scans included filtering, segmentation, calculation of Volume of Interest (VOI), porosity and void volume ^1,2^. The images were filtered using median filter with three iterations and interpretation module set to 3D. The median filter uses morphological operators for setting the voxel values and the filters reduce the contrast in images and soften their edges. The islands filter was applied in 3D volume to fill islands less than 15 voxels. The filtered images were segmented into the target anatomical regions with the help of multi-thresholding segmentation module. Intensity thresholds were applied first to separate air and the regions of the berries, in the histogram. The structures of whole berries were separated from the background and the whole berry, the seed, and the epicarp with sarcocarp were the three separated regions. The volume per VOI were calculated using the material statistics module and linear interpolation of the labelled regions. The void volume and area, and porosities were calculated by using the arithmetic and porosity modules. The processing of images enabled quantification of various morphological structures, the values obtained from the image analysis modules were statistically compared by Analysis of Variance (ANOVA) using Instat Graphpad Software.

**S33 Sample application and instrument settings for MALDI-TOF/TOF-MS analysis.**

For five samples of each variety, triplicates were spotted (1 μL each) on an AB Sciex 384-Opti-TOF plates (123 x 81 mm). Briefly, 5, 10, 15 and 20 μL of each of the extracts (10 mg/mL) in chloroform: methanol (1:1 v/v) were diluted to 200 μL with isopropanol:acetonitrile (60:40 v/v). Next, 10 μL of diluted samples were individually mixed with 10 μL of the matrix, 9-amino acridine (10 mg/mL dissolved in isopropanol:acetonitrile 60:40 v/v). External calibration standards were spotted in spots next to the samples.

The following instrument parameters were used in negative mode: laser pulse rate 404 Hz, laser intensity 5700 on a fixed mode, 50 total shots per spectrum and 10 shots per spectrum. Raw spectrum filtering and peak detection were carried out with S/N threshold of 20 with AB Sciex Data Explorer software (version 4.11, 2010). All spectra obtained from MALDI-TOF/TOF MS analysis of extracts of berries were pre-processed to reduce the background interference. All spectra were normalized to a S/N value of 20 with AB Sciex Data Explorer software (version 4.11, 2010). The observed *m/z* values of the identified compounds and their intensities were extracted. The identities of the detected compounds were confirmed by comparing the experimental *m/z* values with values from literature and METLIN database with a mass tolerance of 50 ppm. The mass measurement accuracy values were calculated in Δppm and were used for dependability on putative identification of the metabolites^3^. The assignments that agreed to previously reported secondary metabolites in the literature and chemical databases were given preferred selection.

**S34 Data processing for MALDI-MSI.**

For imaging experiments, the mass spectra and images were visualized and processed by MSiReader (v0.09) and Matlab (version R2016b)^4^. A mass window of 50 ppm was used for analyses of all selected peaks, without normalization. Peaks of interest were selected with their respective *m/z* values, with a linear interpolation of order 0. Colocalization of processed images was carried out with the RGB colocalization feature of MSiReader.

**S35 Laser microdissection, GC-MS and LC-MS analysis.**

**Laser microdissection of samples:** The preparation and laser microdissection of specific tissues of both varieties of samples were carried out as per the procedure detailed in our previous studies ^5^. The samples were softened for sectioning by placing under vacuum at 25 inHg at room temperature for 24 hrs. Prior to being subjected to vacuum the samples were wrapped in non-cellulose paper soaked in ultrapure water. The softened samples were cut and embedded in cryogel matrix (Leica Microsystems, Germany) and then sectioned with a cryostat at -15°C. The sections were cut at a thickness of 15 μm and mounted on polyethylene terephthalate slides using a Leica CM 1850 cryostat. For laser dissection of target tissues, a Zeiss Palm Microbeam system (P.A.L.M. Microlaser Technologies AG, Germany) equipped with a PALMRobo (Version:V4.6.0.4) software, was used. The tissues were dissected at 5x magnification with delta values for energy and focus at 25 and 96, respectively. The exposure time was 110 ms, and laser energy of 70. The sectioned tissues were collected in Eppendorf tube caps prefixed in the PALMRobo device with a total area of about 1x 10^6^ μm^2^.

**GC-MS and LC-MS analysis:** For gas chromatography–mass spectrometry (GC-MS) analysis, the laser dissected tissues and powders of whole berries were extracted in ethyl acetate: hexane (1:1) and for liquid chromatography–mass spectrometry (LC-MS) the samples were extracted with methanol as the solvent. The samples (0.5 g/mL) were extracted for 30 min with ultrasonication (Elma, Elmasonic P30H) in respective solvents, at room temperature. After sonication, the samples were centrifuged at 12000 rpm for 10 min and the resultant supernatants were filtered and used for further processing and analysis. The standard compounds, myristic acid, 9-octadecenoic acid, delta-tocopherol, gamma tocopherol and palmitic acid were purchased from Sigma. Dodecanoic acid was purchased from CRS (Cedex, Strasbourg). All other reagents and solvents used were of mass spectrometry grade.

For GC-MS analysis, the samples were dried under flow of nitrogen gas. After drying, the samples and standards were derivatized with N-methyl-N-(trimethylsilyl) trifluoroacetamide (MSTFA) + 1% trimethylchlorosilane (TMCS) as the derivatizing agents. The mixtures of samples and the derivatizing agents were incubated at 70°C for 60 min. The samples were cooled before analysis. An Agilent 7890A GC system, with an Agilent 5975C electron ionization (EI) mass selective detector (MSD) was used. It was equipped with DB-5MS capillary column (30 m length, 250 μm i.d., 0.25 μm film thickness). Helium was used as the carrier gas with a constant flow rate of 1 mL min^−1^. The system was operated in a split mode and the GC oven program was set with initial temperature of 70°C held for 1 min. The temperature was then ramped up to 160°C at the rate of 30°C min^-1^ and held at 1min, then ramped up to 280°C at the rate of 10°C min^-1^ and held for 1 min. The transfer interface temperature was maintained at 270°C and the retention times of characteristic ions were recorded using electron ionization mass spectra generated in scan mode (mass range *m/z* 45-600). The GC-MS data were exported from the instrument computer in .aia format and processed using LECO ChromaTOF software, version 4.51.6.0 (Leco Corporation, St. Joseph, MI). Baseline smoothing, peak picking, automated and manual peak identification and peak integration were performed. All automated integrations were manually interrogated and corrected, if necessary. The results of quantitation for selected standards is an average of five samples selected for each variety of berries.

For LC-MS analysis, a UPLC-QTOF MS system (ACQUITY UPLC-Quattro Premier XE MS, Waters Corp., Milford, MA) was used. A UPLC C_18_ analytical column [dimensions 2.1 × 100 mm, i.d. 1.8 μm, Acquity (BEH), Waters, MA] was used for separation. Mass spectra were acquired in both positive and negative electrospray ionization mode. The mobile phase consisted of 0.1% formic acid in water (solvent A) and 0.1% formic acid in acetonitrile (solvent B). The gradient used for analysis was: 0-1.0 min (1% B), 1.0-3.0 min a linear increase from 15 to 50% B, 3.0-8.0 min at 50-85% B, 8.0-10.0 min at 85-100% B, 10.0-13.0 min at 100% B, and a decrease from 100-1% B at 13-13.5 mins followed by further decrease to 1% B from 13.5-15.0 min. The desolvation temperature was maintained at 350°C and the capillary voltage was set to 3.2 kV. Leucine enkephalin was used as a lock mass standard and p-Chlorophenylalanine (75 μg/mL) was used as an internal standard. The raw data files generated by UPLC-QTOF-MS were processed using Progenesis QI software (Waters Corp., Milford, MA). The data were normalized to the added internal standards and putative metabolite annotations were carried out based on the molecular ions of each detected signal from the study samples and the masses reported in literature.

**References:**

1. Musse, M. *et al.* Quantification of microporosity in fruit by MRI at various magnetic fields: Comparison with X-ray microtomography. *Magn. Reson. Imaging* **28,** 1525–1534 (2010).

2. Herremans, E. *et al.* Automatic analysis of the 3-D microstructure of fruit parenchyma tissue using X-ray micro-CT explains differences in aeration. *BMC Plant Biol.* **15,** 264 (2015).

3. Li, B., Bhandari, D. R., Römpp, A. & Spengler, B. High-resolution MALDI mass spectrometry imaging of gallotannins and monoterpene glucosides in the root of Paeonia lactiflora. *Sci. Rep.* **6,** 36074 (2016).

4. Robichaud, G., Garrard, K. P., Barry, J. A. & Muddiman, D. C. MSiReader: An open-source interface to view and analyze high resolving power MS imaging files on matlab platform. *J. Am. Soc. Mass Spectrom.* **24,** 718–721 (2013).

5. Jaiswal, Y. *et al.* Distribution of toxic alkaloids in tissues from three herbal medicine Aconitum species using laser micro-dissection, UHPLC-QTOF MS and LC-MS/MS techniques. *Phytochemistry* **107,** 155–174 (2014).
