# Supplementary figures and images for: A substitute variety for agronomically and medicinally important Serenoa repens (saw palmetto)
Source: Sci Rep. 2019 Mar 18;9:4709. doi: 10.1038/s41598-019-41150-z (PMC6423146; doi:10.1038/s41598-019-41150-z)

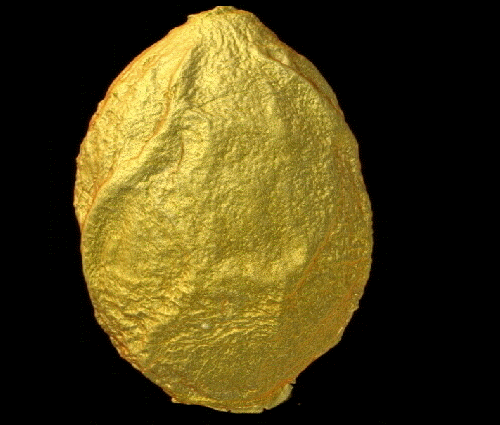

Supplement: Supplementary file 2 — Supplementary Movie S1 [file 41598_2019_41150_MOESM2_ESM.gif]

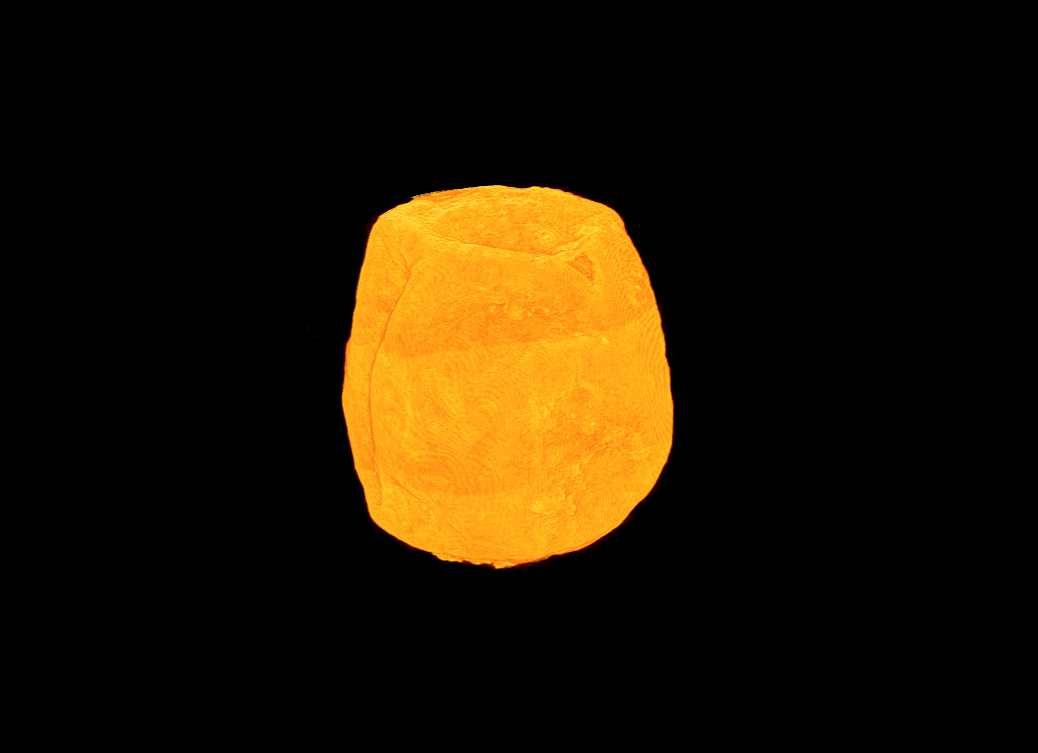

Supplement: Supplementary file 3 — Supplementary Movie S2 [file 41598_2019_41150_MOESM3_ESM.gif]
